# Atomic‐Level Interface Engineering for Boosting Oxygen Electrocatalysis Performance of Single‐Atom Catalysts: From Metal Active Center to the First Coordination Sphere

**DOI:** 10.1002/advs.202205031

**Published:** 2022-11-23

**Authors:** Qizheng An, Shuowen Bo, Jingjing Jiang, Chen Gong, Hui Su, Weiren Cheng, Qinghua Liu

**Affiliations:** ^1^ National Synchrotron Radiation Laboratory University of Science and Technology of China Hefei Anhui 230029 P. R. China; ^2^ Institute for Catalysis Hokkaido University Sapporo 001‐0021 Japan

**Keywords:** active center, atomic interface modification, electrocatalysis, single‐atom catalysts, the first coordination sphere

## Abstract

Oxygen reduction reaction (ORR) and oxygen evolution reaction (OER) are the core reactions of a series of advanced modern energy and conversion technologies, such as fuel cells and metal–air cells. Among all kinds of oxygen electrocatalysts that have been reported, single‐atom catalysts (SACs) offer great development potential because of their nearly 100% atomic utilization, unsaturated coordination environment, and tunable electronic structure. In recent years, numerous SACs with enriched active centers and asymmetric coordination have been successfully constructed by regulating their coordination environment and electronic structure, which has brought the development of atomic catalysts to a new level. This paper reviews the improvement of SACs brought by atom‐level interface engineering. It starts with the introduction of advanced techniques for the characterizations of SACs. Subsequently, different design strategies that are applied to adjust the metal active center and first coordination sphere of SACs and then enhance their oxygen electrocatalysis performance are systematically illustrated. Finally, the future development of SACs toward ORR and OER is discussed and prospected.

## Graphical abstract

This review classifies different design strategies applied in metal active center and the first coordination sphere, including engineering dual‐atom and triple‐atom sites, changing coordination atom species, doping the heteroatoms, and achieving spatial coordination structure. Moreover, advanced characterization techniques are introduced, and future developments of single‐atom catalysts toward oxygen reduction reaction and oxygen evolution reaction are prospected.

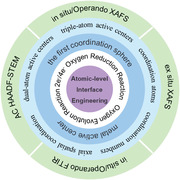

## 1 Introduction

The massive consumption of fossil fuels results in serious environmental problems and huge impact on human existence and social stability. In order to mitigate these problems, some efficient energy conversion technologies are urgently needed, such as thermal catalysis, photocatalysis, and electrocatalysis. Among the above three kinds of technologies, electrocatalysis has received extensive attention from the scientific and industry communities owing to their mild working environment, easy operation, and promising catalytic performance.^[^ ^1^, ^2^, ^3^ ^]^ Electrocatalytic processes generally involved oxygen, including oxygen reduction reaction (ORR) and oxygen evolution reaction (OER), are central to a series of advanced modern energy and conversion technologies, for example, water electrolyzers, fuel cells, metal–air batteries, and proton exchange membrane fuel cells. However, the intrinsically sluggish 4‐electron transfer process of ORR and OER as well as the poor stability of catalysts under operating conditions has become the bottlenecks for large‐scale applications of these devices.^[^ ^4^, ^5^ ^]^ Therefore, toward meeting the increase of global energy demands and the urgent requirement of a cleaner and pollution‐free environment, it is imperative to develop advanced catalysts with excellent performance, robust stability, and effective cost.^[^ ^6^ ^]^

Previous studies have indicated that ORR and OER activity of catalysts is highly dependent on the density and chemical properties of active sites. Compared to bulk materials, ultrasmall particles possess larger surface area, unsaturated‐coordination environment, and strong metal–support interactions.^[^ ^7^, ^8^ ^]^ These features contribute to the increase of the quantity of active sites and enhanced interplay between supports and metal atoms. When the size of active nanoparticle is ultimately reduced into the atomic scale (even single atoms), maximum atomic utilization and superior catalytic performance will be obtained for catalysts.^[^ ^9^ ^]^ In 2011, Zhang and co‐workers developed Pt_1_/FeO*_x_* catalysts with atomically dispersed active sites, achieving excellent CO oxidation activity and stability.^[^ ^10^ ^]^ This was the first time for the concept of single‐atom catalysts being put forward. Their research results promote single‐atom catalysts (SACs) into blossoming development, and make SACs become the most popular catalysts in the past decade. With undercoordinated nature, single atom can be coordinated, embedded, adsorbed, chemisorbed onto a high‐surface‐area solid support as advanced SACs. Moreover, the unique geometric and electronic properties of SACs, for example, the absence of metal–metal bonds and positively charged single metal atoms, are beneficial for significantly altering the adsorption properties and binding energies of intermediates over their metal centers.^[^ ^11^ ^]^ These unique advantages endow SACs with remarkable catalytic activity and selectivity, and create the potential for them to be widely employed in many electrocatalytic reactions, including ORR,^[^ ^12^, ^13^, ^14^ ^]^ OER,^[^ ^15^, ^16^, ^17^ ^]^ hydrogen evolution reaction (HER),^[^ ^18^, ^19^, ^20^ ^]^ CO_2_ reduction reaction (CO_2_RR),^[^ ^21^, ^22^, ^23^ ^]^ and nitrogen reduction reaction (NRR).^[^ ^24^, ^25^, ^26^ ^]^

SACs, especially those supported on N‐doped porous carbon (M—N*_x_*—C), have produced a large number of promising results. However, with the continuous development of electrocatalysis, the performance and the industrial application potential of these catalysts are increasingly being taken seriously. Some disadvantages of SACs are beginning to expose. 1) Symmetrically coordinated SACs seem unable to meet the optimal adsorption of intermediates. Previous research showed that the standard symmetrical planar four coordinated structure (denoted as M—N_4_ moiety) might serve as the most favorable catalytic site for M—N*_x_*—C catalysts.^[^ ^27^, ^28^, ^29^, ^30^ ^]^ However, some recent research work pointed out that the large electronegativity of symmetrical adjacent nitrogen atoms around the metal site in M—N_4_ moiety would result in inappropriate free energy of adsorbed intermediates.^[^ ^31^, ^32^ ^]^ Obviously, it will seriously reduce the kinetic activity of the catalysts and weaken their performance. 2) In principle, SACs are not competent for reactions whose pathways require synergetic interactions between two or more adjacent active metal atoms.^[^ ^33^ ^]^ Hence, the single active sites of SACs are difficult to effectively regulate the reaction path. For example, due to the lack of continuous active sites, Pt single atom catalysts generally tend to transfer 2e^−^ to produce H_2_O_2_ rather than generate H_2_O through 4e^−^ pathway, severely reducing the selectivity toward target products.^[^ ^34^ ^]^ It is well‐known that the reaction model, reaction path, stability, and intrinsic activity of catalysts are closely associated with the metal active centers and their coordination environment. Therefore, adjusting the structure of active sites is the fundamental method of breaking these limitations.

**Figure 1:**
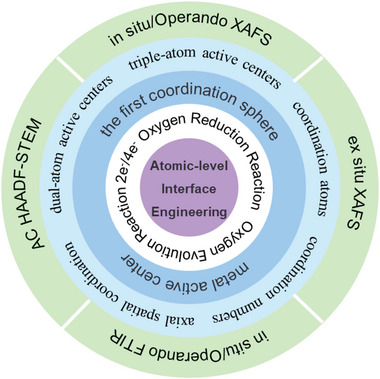
An overview of the topics involved in this review.

Summarizing previous research, we found that the effective tailoring for SACs mainly focused on enriching the types of active center and tuning the coordination environment of active centers, which is closely associated with the active centers and their first coordination sphere. In this review, we refer to the above improvement of the active center and coordination environment of SACs as atom‐level interface modification engineering. Especially, the popular design strategies of advanced SACs toward 2e^−^/4e^−^ ORR and OER in recent years will be systematically illustrated. As shown in **Figure** **1**, it will start with the introduction of advanced characterization techniques of SACs. Subsequently, we focus on the different design strategies for regulating the interface configuration of SACs in the active center and the first coordination sphere. To distinguish the type of metal atoms, adjacent coordination dopants, and the coordination number (CN) can help us investigate the nature of single‐atom active sites and greatly enrich our understandings of their catalytic activity and selectivity. When it comes to the design of the active center, we will detail the construction of dual‐atom and triple‐atom active centers that have triggered great interest in recent years. As far as we know, this is the first time that the development of triple‐atom site catalysts toward efficient ORR and OER has been systematically summarized. Whereas in the optimization of the first coordination sphere, the influences of coordination atoms, coordination numbers, and axial spatial coordination structures of SACs will be illustrated. At the end of this review, the prospects and challenges of SACs in the future will be proposed. It is believed that this review could bring inspiration for the design and evolution of atomic catalysts in material science and electrocatalysis communities.

## 2 Characterization Techniques

The identification of structure configurations of metal centers in SACs, such as the chemical environment, the coordination atom, and coordination number, is prerequisite for determining the successful synthesis. Therefore, advanced and accurate characterization methods are indispensable for establishing a clear connection between the structure and property for SACs.^[^ ^35^ ^]^ With the development of characterization techniques, characterization approaches are not limited to X‐ray diffraction, transmission electron microscopy (TEM), X‐ray photoelectron spectroscopy (XPS), Fourier transform infrared spectroscopy (FTIR), Raman spectroscopy, and nuclear magnetic resonance.^[^ ^36^ ^]^ Researchers could have a more comprehensive and in‐depth understanding of the structure of catalytic sites and their dynamic evolution during the reaction process by using aberration correction high‐angle annular dark field scanning transmission electron microscopy (AC HAADF‐STEM), X‐ray absorption fine structure spectroscopy (XAFS), and in situ/operando XAFS and FTIR.

### 2.1 AC HAADF‐STEM

**Figure 2:**
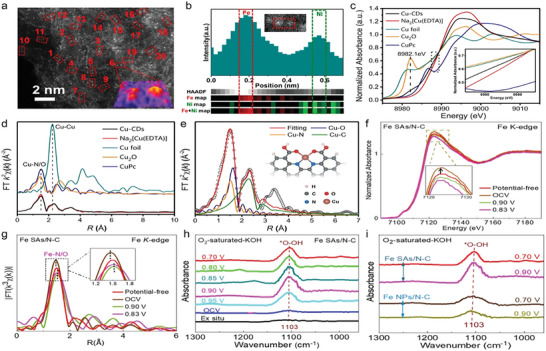
a) Aberration‐corrected HAADF‐STEM image of Fe—Ni—N—C and b) simultaneously acquired HAADF‐STEM image intensity profile accompanied by atomic‐resolution EELS mapping of the Fe—Ni pair presented in panel (d). Reproduced with permission.^[^ ^38^ ^]^ Copyright 2021, American Chemical Society. c) XANES spectra and d) EXAFS spectra at the Cu K‐edge of Cu‐CDs, Na_2_[Cu(EDTA)](EDTA is short for ethylene diamine tetraacetic acid), Cu foil, Cu_2_O, and CuPc samples. e) EXAFS fitting curves of Cu‐CDs in *R* space using backscattering paths of Cu—N, Cu—O, and Cu—C. Reproduced with permission.^[^ ^39^ ^]^ Copyright 2021, Springer Nature. f) The operando XANES spectra and g) the operando EXAFS *R*‐space spectra of the Fe SAs sample under different voltage conditions. h) 960–1300 cm^−1^ range of operando FTIR characterizations under different ORR working potentials for Fe SAs/N—C. i) 960–1300 cm^−1^ range of FTIR signal under two typical potentials for the Fe SA sample and the Fe NP sample. Reproduced with permission.^[^ ^46^ ^]^ Copyright 2021, American Chemical Society.

With ultrahigh atomic resolution (0.1 nm), AC HAADF‐STEM provides atomic‐scale structural information of underlying active sites and their metal–support interactions for SACs. It could clearly observe the distribution and position of single and dual atom sites, providing direct evidence for the existence of atomic sites. In addition, AC HAADF‐STEM can be employed to perform linear and planar scanning of energy dispersive spectroscopy and electron energy loss spectroscopy (EELS) to discover the chemical elements of SACs, which helps to speculate the possible coordination relationship between atomic sites and different elements.^[^ ^37^ ^]^ For instance, AC HAADF‐STEM detection for Fe—Ni—N—C catalysts demonstrated that atomically dispersed atoms were in the form of atom pair (**Figure** **2**a). Considering the brightness of the metal with similar atomic number is hard to tell apart in the HAADF‐STEM image, EELS technique was employed to identify the elemental composition in dual‐atom pair. As shown in Figure 2b, it is easy to find that each atom pair consisted of one Fe atom and one Ni atom.^[^ ^38^ ^]^

### 2.2 XAFS

XAFS analysis can provide more valuable information about the geometric and electronic structures of the active sites in SACs. In general, XAFS could be divided into X‐ray absorption near‐edge structure (XANES) and extended X‐ray absorption fine structure (EXAFS) spectra. The former contains energies from the absorption edge to 30–50 eV higher than the absorption edge, which can provide information of the geometric structure and chemical environment of the active sites. While the latter extends the energy range of the absorption edge from 30–50 to 1000 eV or more. EXAFS offers the local bond information of the central metal atom in the form of weighted average to determine the average coordination number as well as the average atomic distance between the absorbing atom and the backscattering atom. Cai et al. deepened the understanding of the coordination environment in Cu SAC (denoted as Cu‐CDs) by virtue of XANES and EXAFS. In Figure 2c, the absorption edge of Cu‐CDs was located between that of Cu(II) phthalocyanine (CuPc) and Cu_2_O, suggesting that the metal valence of Cu species was between +1 and +2. For the EXAFS spectra (Figure 2d), Cu‐CDs displayed a prominent peak at 1.5 Å, corresponding to Cu—O/N bond. Moreover, there were no Cu—Cu coordination peaks at 2.2 Å, implying the single atom states of Cu species. EXAFS fitting results illustrated that the Cu center was coordinated with double N atoms and double O atoms, as shown in Figure 2e, and the structure of Cu‐CDs could be determined to Cu—N_2_O_2_ (structural model is in the inset).^[^ ^39^ ^]^

### 2.3 In Situ/Operando XAFS and FTIR

The operando X‐ray absorption spectroscopy has been widely conducted on SACs to unveil the dynamic evolution of the electronic structure and geometric environment of the atomic metal sites during catalytic process;^[^ ^40^ ^]^ whereas operando FTIR has the access to capture the dynamic behaviors of intermediates under realistic operating conditions.^[^ ^41^ ^]^ The combination of in situ XAFS and in situ FTIR techniques could help us monitor the interfacial evolution of the active site and get a more thorough understanding of the action mechanism.^[^ ^42^, ^43^, ^44^, ^45^ ^]^ In order to identify the dynamic evolution of Fe active sites within Fe—N—C SACs, operando XAFS characterization was carried out. As shown in Figure 2f, with gradually applying oxygen reduction driven voltage, the white line peak intensity at Fe K‐edge decreased (from open‐circuit voltage to 0.83 V vs reversible hydrogen electrode (RHE)), which indicated the changes of electronic structure for Fe active sites. The redistribution of the electronic orbital configuration suggested a dynamic evolution of the coordination environment at Fe sites occurring during the catalytic operation. This was further determined by operando EXAFS in Figure 2g. The dominant peak located at 1.5 Å corresponded to the main Fe—N/O coordination path of Fe sites. Changes in peak intensities with voltage varying implied the variation of the local structure around Fe centers. EXAFS‐fitting analysis showed that under the open‐circuit voltage condition, an additional Fe—O coordination appeared in the form of HO—Fe—N_4_, implying the OH was preadsorbed on Fe site to activate its electronic structure. What is noteworthy is that when the potential declined to 0.90 V, the HO—Fe—N_4_ was first released dynamically as OH—Fe—N_2_ with two Fe—N bonds being broken, followed by the formation of an additional Fe—O coordination. Coordination‐unsaturated OH—Fe—N_2_ active site was more likely to promote the breaking of the O—O bond of *OOH intermediates. At real‐time working condition, the dynamic behavior of *OOH intermediates was tracked via operando FTIR techniques (Figure 2h). The absorption bands at 1103 cm^−1^ were observed after the ORR potential applied, pointing to the accumulation of the crucial *OOH intermediate at the HO—Fe—N_2_ active sites. Compared with the almost negligible changes in Fe NPs (Figure 2i), the absorption intensity of *OOH intermediate in Fe SACs displayed an evident decline that meant the cleavage of O—O bond in *OOH, thus accelerating the ORR process.^[^ ^46^ ^]^

## 3 Modification Engineering in the Active Center

### 3.1 Dual‐Atom Active Center

There are a large number of excellent studies on SACs that have been reported in the past decade. However, the inherent defects of SACs are still hard to cover up. Their relatively low loading mass of metal sites and single‐atom site nature render them difficult to effectively regulate reaction pathways involving multiple reaction intermediates/products. As a promising strategy to break these limitations, engineering dual‐atom site catalysts (DASCs) has become the research hotspots in recent years.^[^ ^47^, ^48^, ^49^, ^50^, ^51^ ^]^ Summarizing previous researches, DASCs could be classified into four categories according to their atom type and configuration: 1) homologous dual single‐atom site catalysts, 2) heterogeneous dual single‐atom site catalysts, 3) homologous dual‐atom pair catalysts, and 4) heterogeneous dual‐atom pair catalysts. The first two catalysts contain two kinds of metal single atom sites, and the distance between them is a bit large. Thus, the interaction between double sites is ambiguous. By contrast, the two atom sites of dual‐atom pair catalysts are in proximity and bond to each other. Strong synergistic effect between them was activated and then the catalytic performance could be further reinforced. Hence, we will place emphasis on the latter two catalysts in this section (the performance of DASCs is summarized in **Table** **1**).

**Table 1:** The oxygen electrocatalysis performance on DASCs

Catalyst	Active center	Media	Performance (@10 mA cm^−2^)	Reference
Fe_2_—N—C	Fe—Fe—N	0.1 m HClO_4_	*E*_1/2_ = 0.78 V	[52]
Fe_2_—N—C	Fe—Fe—N	0.1 m KOH	*E*_1/2_ = 0.905 V	[52]
Fe_2_N_6_	Fe—Fe—N	0.5 m H_2_SO_4_	*E*_1/2_ = 0.84 V	[53]
Fe_2_@PDA—ZIF‐900	Fe—Fe—N	0.5 m H_2_SO_4_	*E*_1/2_ = 0.816 V	[54]
Fe_2_@PDA—ZIF‐900	Fe—Fe—N	0.1 m KOH	*E*_1/2_ = 0.951 V	[54]
Fe_2_—GNCL	Fe—Fe—N	1 m KOH	*E*_over_ = 0.355 V	[55]
(Fe,Co)/N—C	Fe—Co—N	0.1 m HClO_4_	*E*_1/2_ = 0.863 V	[56]
(Fe,Co)/CNT	Fe—Co—N	0.1 m KOH	*E*_1/2_ = 0.954 V	[57]
Zn/CoN—C	Zn—Co—N	0.1 m KOH	*E*_1/2_ = 0.861 V	[58]
a‐NiCo/NC	Ni—Co—N	1 m KOH	*E*_over_ = 0.252 V	[59]
NiFe DASC	Ni—Fe—N	1 m KOH	*E*_over_ = 0.31 V	[60]
Ru—Co/LCO	Ru—Co—N	1 m KOH	*E*_over_ = 0.247 V	[61]
FeNi SAs/NC	Ni—Fe—N	1 m KOH	*E*_over_ = 0.27 V	[62]
Fe—NiNC	Ni—Fe—N	1 m KOH	*E*_over_ = 0.34 V	[63]
IrCo—N—C	Ir—Co—N	0.1 m KOH	*E*_1/2_ = 0.911 V	[64]
IrCo—N—C	Ir—Co—N	1 m KOH	*E*_over_ = 0.33 V	[64]
CoNi SAs/NC	Ni—Co—N	0.1 m KOH	*E*_1/2_ = 0.76 V	[65]
CoNi SAs/NC	Ni—Co—N	1 m KOH	*E*_over_ = 0.34 V	[65]

#### 3.1.1 Homologous Dual‐Atom Active Center

**Figure 3:**
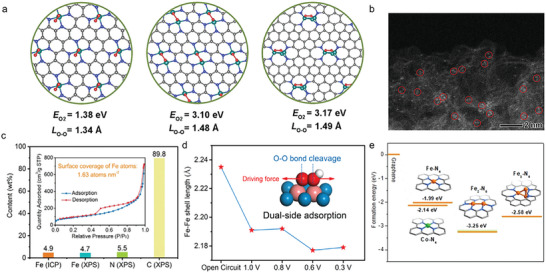
a) Superoxo‐like adsorption model at Fe_1_—N—C, peroxo‐like adsorption model at Fe_2_—N—C, and peroxo‐like adsorption model at Fe_3_—N—C. b) HAADF‐STEM images of Fe_2_—N—C. Reproduced with permission.^[^ ^52^ ^]^ Copyright 2019, Elsevier. c) Elemental content of planar‐like Fe_2_N_6_ structure, obtained from XPS and inductively coupled plasma‐atomic emission spectrometry measurements. d) Fe—Fe shell length calculated from operando EXAFS spectra. Inset: deductive oxygenated intermediates adsorption state on the planar‐like Fe_2_N_6_ structure. Reproduced with permission.^[^ ^53^ ^]^ Copyright 2020, Elsevier. e) Formation energies of Fe/Co—N_4_, Fe_2_—N_6_, and Fe_3_—N_4_ moieties from graphene. Reproduced with permission.^[^ ^55^ ^]^ Copyright 2020, John Wiley and Sons.

“Precursor‐preselected” strategy was widely used in preparing homologous dual‐atom pair catalysts. Preselected metal complexes with dual‐atom structures can direct the dual‐atom sites to anchor on the substrate materials. In 2019, Ye et al. prepared Fe*_n_* clusters (**Figure** **3**a) with the accurate atom number (*n* = 1–3) via employing Fe(acac)_2_@ZIF (zeolitic imidazolate framework)‐8, Fe_2_(CO)_9_@ZIF‐8, and Fe_3_(CO)_12_@ZIF‐8 as precursors, respectively. Among them, Fe_2_ cluster (dual‐atom site can be seen in Figure 3b) exhibited the best acidic (*E*_1/2_ = 0.78 V) and alkaline (*E*_1/2_ = 0.905 V) ORR activity compared with Fe_1_—N—C (0.715/0.887 V) and Fe_3_—N—C (0.762/0.891 V). This excellent performance resulted from unique peroxo‐like oxygen adsorption model that could possess higher adsorption energy. In addition, the graphitization of N‐doped carbon as well as the controllable N species accelerated the electron transport during ORR process.^[^ ^52^ ^]^ Zhang et al. constructed high‐density Fe_2_—N_6_ catalysts through pyrolytic migration method. In Figure 3c, the Fe content was as high as 4.9 wt% determined by inductively coupled plasma‐atomic emission spectrometry (ICP‐AES), implying a high coverage of Fe_2_N_6_ sites on the carbon surface. There was also an optimized oxygen adsorption structural model for Fe_2_—N_6_ catalysts, as shown in Figure 3d. The two adjacent Fe atoms in Fe_2_—N_6_ bind to an O atom of the oxygen‐containing intermediate separately, forming the special dual‐side adsorption and providing a strong driving force for O—O bond breaking. Therefore, the dissociation process of oxygen intermediate was promoted.^[^ ^53^ ^]^ Leng et al. discovered that Fe—Fe atom pair had less antibonding orbital filling and more positive average d‐band center relative to that of single atom Fe catalyst, which could facilitate electron transfer to oxygen and then activate the O—O bond.^[^ ^54^ ^]^

Fe_2_ microclusters with distinguished OER performance were reported by Xu and co‐workers. They encapsulated the trinuclear Fe^III^_2_Fe^II^ complex in a metal–organic framework (MOF), and tailored target catalyst by substituting Zn atoms for partial Fe^II^ atoms. During the sintering, Zn metal species evaporated and the Fe_2_—N—C catalysts were obtained. Thermodynamic evidence (Figure 3e) suggested that the Fe_2_—N—C structure was stable, where dual‐atom Fe atoms were stabilized in an optimal M—N moiety within the carbon layer as efficient OER catalysis sites.^[^ ^55^ ^]^

#### 3.1.2 Heterogeneous Dual‐Atom Active Center

**Figure 4:**
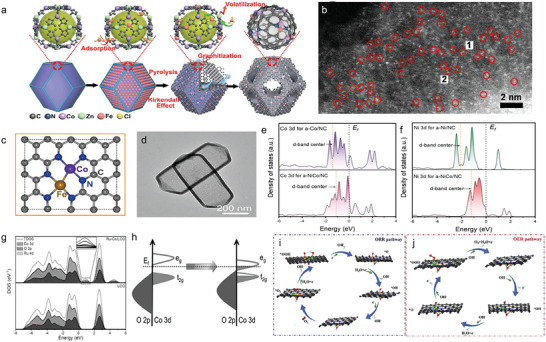
a) Schematic illustration for the preparation of Fe—Co DACs. b) Magnified HAADF‐STEM images of Fe—Co DACs. c) The proposed architectures of Fe—Co dual sites. Reproduced with permission.^[^ ^56^ ^]^ Copyright 2017, American Chemical Society. d) TEM images of a‐NiCo/NC. e) Calculated PDOS of Co 3d and f) Ni 3d for a‐Co/NC and a‐NiCo/NC. Reproduced with permission.^[^ ^59^ ^]^ Copyright 2022, John Wiley and Sons. g) Total and projected density of states (TDOS and PDOS) Co 3d, O 2p, and Ru 4d DOS. h) Qualitative one‐electron energy diagram. Reproduced with permission.^[^ ^61^ ^]^ Copyright 2022, John Wiley and Sons. i) ORR and j) OER reaction mechanisms. The black, blue, green, golden, red, and pink balls represent C, N, Ni, Fe, O, and H atoms, respectively. Reproduced with permission.^[^ ^63^ ^]^ Copyright 2020, Elsevier.

Owing to the excellent activity of Co toward ORR, Co—M (M = Fe, Zn, etc.) configurations are reckoned as promising DASCs to make ORR performance move forward a step. Wu and co‐workers developed a host–guest strategy to engineer the Fe—Co dual atom catalysts (DACs, **Figure** **4**a). Initial, FeCl_3_ molecule was encapsulated in Zn/Co bimetallic MOF via double solvents methods, which could alleviate the diffusion resistance caused by the narrow aperture of ZIF. During the pyrolysis, Fe—Co atom sites anchored on nitrogen‐doped porous carbon, and they can be clearly observed in HAADF‐STEM image (Figure 4b). Combining the simulated XANES spectra and fitted EXAFS spectra at Fe K‐edge, the coordination environment of Fe—Co DACs was determined (Figure 4c).^[^ ^56^ ^]^ Wu and co‐workers also embedded Fe—Co atom pair into carbon nanotube (CNT), and acquired ultrahigh ORR activity. The half‐wave potential tested in alkaline electrolytes was up to 0.954 V, surpassing the vast majority of reported catalysts.^[^ ^57^ ^]^ Rich in C and N sources, polychitose can be used as the substrate to fix dual‐atom sites as well. Lu et al. designed Zn—Co configuration catalysts via competitive complexation strategy. Taking advantage of the similar coordination ability between Zn and Co, dual metal species could coordinate equally with the —NH_2_ and —OH groups on the polychitosan chain, thus achieving a uniform dispersion of Zn—Co sites. Density functional theory (DFT) unveiled that such architecture offered reinforced binding ability of O_2_ and then could facilitate ORR dynamics greatly.^[^ ^58^ ^]^

The researches on dual‐atom centers with efficient OER catalysis activity collectively reveal the key role of dual‐atom sites on optimizing electron configuration of DASCs. Recently, Lou and co‐workers reported a novel Ni—Co DASC (denoted as a‐NiCo/NC) anchored to a nitrogen‐doped hollow prism (Figure 4d). Its total metal content in a‐NiCo/NC was 1.6 wt% (determined by ICP‐AES), which was almost double that of a‐Ni/NC (0.62 wt%) and a‐Co/NC (0.65 wt%) samples, indicating the superiority of loading than SACs. In alkaline electrolytes, a‐NiCo/NC displayed an ultralow overpotential of 252 mV. The superb activity origin was investigated by theoretical calculation. An obvious density of states (DOS) overlap of Co 3d state and Ni 3d state can be observed from −4 to 2 eV in Figure 4e,f, suggesting the strong electronic coupling between Ni and Co atoms.^[^ ^59^ ^]^ Electronic coupling also existed in Ni—Fe DASC, and the unique electronic state increased the oxidation state of iron and smoothed the binding strength of the intermediate.^[^ ^60^ ^]^ Zheng et al. substituted a Ru atom for a Co site in the layer LiCoO_2_ (the catalyst was named as Ru‐Co/LCO), and investigated the effect on the ligand field and electron configuration of the active center brought by Ru atom. They found that the introduced Ru species would alter the electronic properties of Co and O atoms because the reinforced electron density near the Fermi level was not only induced by the Ru 4d orbital but also by the Co 3d and O 2p orbitals (Figure 4g). In addition, the increasing valence band centers of Co 3d and O 2p and their narrow band center gap indicated the strengthening of the Co—O covalent bond. The evolution of the electron configuration was also investigated. In Figure 4h, the calculated number of e_g_ electrons changed from 0.35 (LCO) to 0.61 (Ru—Co/LCO) that meant the introduction of Ru atom increased the filling of e_g_.^[^ ^61^ ^]^

Oxygen reduction reaction and oxygen evolution reaction are core actions in series of energy storage and conversion devices where the bifunctional activity of catalysts determines their overall energy conversion efficiency. Therefore, developing catalysts with robust bifunctional performance is quite necessary. Yu et al. designed the FeNi single atom (SA)/NC dual‐atom site catalyst whose OER (*E*_over_ = 270 mV) and ORR (*E*_1/2_ = 840 mV) activity were superb. Its practical applications were accessed in Zn–air rechargeable battery. The specific capacity was measured to be 779.4 mAh g^−1^, which can compete with that of Pt/C (696.7 mAh g^−1^). Moreover, the charging–discharging potential gap of FeNi SAs/NC did not increase over 45 h test, verifying the promising cyclic stability.^[^ ^62^ ^]^ Lu and co‐workers developed Fe—Ni DASC as well, and acquired high power density and durable circulation stability when it was integrated into metal–air metal. Dual‐metal sites in Fe—Ni DASC played their respective roles in ORR and OER. During the ORR process, O atom originated from the cleavage of O—O bond was prone to adsorb on Fe sites due to the lower formation energy (Figure 4i). Whereas in OER cycle, all intermediates were adsorbed on Ni sites because of their greater activity toward OER (Figure 4j).^[^ ^63^ ^]^ Chen and co‐workers discovered that the addition of Ir could induce the electron rearrangement in Co d orbitals as well as enhanced spin polarization and electron transport from oxygen. Therefore, the reaction kinetics reaction kinetics was accelerated.^[^ ^64^ ^]^ Han et al. obtained Co—Ni dual‐atom catalysis through pyrolysis of dopamine‐coated metalorganic frameworks. The synergistic interaction between adjacent dual‐metal sites optimized the adsorption of the key intermediate OH* and effectively reduced the reaction energy barrier for ORR and OER.^[^ ^65^ ^]^

### 3.2 Triple‐Atom Active Center

Encouraged by the successful development of dual‐atom site catalysts, it is easy to think of extending active sites to three‐atom site combinations. The triple‐atom site catalysts (TASCs) have the smallest hollow site as well as the better adsorption condition for surface species.^[^ ^66^ ^]^ Furthermore, the increase of the number of atoms enriches the spatial geometric configuration of the active center and the theoretical loading mass of metal sites, which contributes to the further enhancement of the catalytic performance. Up to now, there have been some studies realizing the application of TASCs in electrocatalysis, photocatalysis, and thermal catalysis,^[^ ^67^, ^68^, ^69^, ^70^ ^]^ and this will undoubtedly trigger wider research interest in the future (the performance of TASCs is summarized in **Table** **2**).

**Table 2:** The oxygen electrocatalysis performance on TASCs

Catalyst	Active center	Media	Performance (@10 mA cm^−2^)	Reference
Co@Pd—Pt	Pt_3_—N	0.1 m KOH	*E*_1/2_ = 0.88 V	[71]
Fe_3_—N—C	Fe_3_—N	0.1 m HClO_4_	*E*_1/2_ = 0.762 V	[52]
Fe_3_—N—C	Fe_3_—N	0.1 m KOH	*E*_1/2_ = 0.891 V	[52]
CTGU‐10c2	Co_2_Ni—N	1 m KOH	*E*_over_ < 0.3 V	[72]
NNU‐23	Fe_2_Ni—N	1 m KOH	*E*_over_ = 0.365 V	[73]
Ni_1_Fe_2_—MOF	Fe_2_Ni—N	1 m KOH	*E*_over_ = 0.355 V	[74]
Ni_1_Fe_2_—MOF	Fe_2_Ni—N	0.1 m KOH	*E*_1/2_ = 0.766 V	[74]

**Figure 5:**
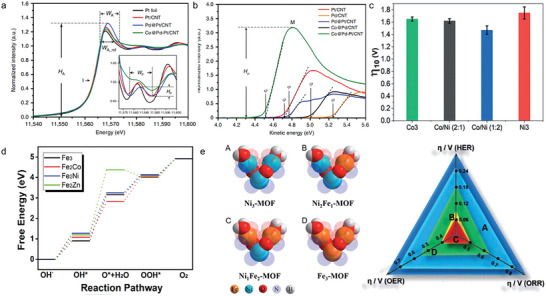
a) XANES of Pt L_3_‐edge of standard Pt foil, Pt/carbon nanotube (CNT), Pd—Pt core–shell/CNT (Pd@Pt/CNT), and Pt‐decorated Co—Pd/CNT (Co@Pd—Pt/CNT) catalysts. The inset shows the enlarged postedge region. b) Ultraviolet photoelectron spectra of Pt‐decorated Co—Pd and controls. Reproduced with permission.^[^ ^71^ ^]^ Copyright 2019, Springer Nature. c) Comparison of the overpotential at a current density of 10 mA cm^−2^. Reproduced with permission.^[^ ^72^ ^]^ Copyright 2019, John Wiley and Sons. d) The free energy profile for the OER pathway. Reproduced with permission.^[^ ^73^ ^]^ Copyright 2018, John Wiley and Sons. e) Visual representation of the V‐shaped trinuclear clusters used in the catalytic model (left). And the theoretical overpotential (*ȵ*) of the HER/OER/ORR as descriptors of trifunctional electrocatalytic activity, and the theoretical overpotential depending on the RDS in DFT (right). Reproduced with permission.^[^ ^74^ ^]^ Copyright 2021, The Royal Society of Chemistry.

Dai et al. decorated low loading (2.4 wt%) platinum trimers on Co@Pd core–shell structure. Their detailed structure was investigated by XAFS analysis. As shown in **Figure** **5**a, the absorption intensity of Co@Pd—Pt catalyst displayed a decrement compared to Pt/CNT, and Pd@Pt/CNT, suggesting a strong charge localization from the surface of Co@Pd to Pt species. This was also determined by ultraviolet photoelectron spectroscopy (Figure 5b), in which the significant enhancement of the M peak near the Fermi level implied that the strong charge localization resulted from the advantage of quantum size effect in Pt_3_ species.^[^ ^71^ ^]^ In the research of Xiong and co‐workers, Fe_2_—N—C catalysts possessed the best ORR activity in both acidic (*E*_1/2_ = 0.78 V) and alkaline (*E*_1/2_ = 0.905 V) electrolyte. However, it should not be ignored that the performance of Fe_3_—N—C (0.762/0.891 V) was comparable to that of Fe_2_—N—C, and outperformed Fe_1_—N—C catalysts (0.715/0.887 V). Just like Fe_2_—N—C, Fe_3_—N—C tended to perform peroxo‐like oxygen adsorption for facilitating the ORR process.^[^ ^52^ ^]^

The studies of TASCs on OER catalysis seem to be more systematic. Bu and co‐workers designed a series of hierarchical bimetallic MOF (named as CTGU‐10a2‐d2). Their metal center composition corresponded to Co_3_, Co_2_Ni, CoNi_2_, and Ni_3_ triple‐atom sites, respectively. Among them, CTGU‐10c2 has shown the best OER performance (Figure 5c) and robust stability over 50 h. DFT revealed that Co was the real origin of activity, whereas the introduction of Ni resulted in the distortion, and shifted the center of the d‐band in Co to a higher energy level.^[^ ^72^ ^]^ A sequence of Fe_2_M (M = Fe, Co, Ni, Zn) cluster catalysts was developed by Lan and co‐workers. DFT unveiled that the adsorption of O* on Fe sites was weak (Figure 5d), resulting in a rather high overpotential for the formation of O*. When a second metal (especially the Ni species) was introduced, d‐band of Fe would approach to the Fermi level, which could enhance the interaction between the intermediate and the active center. Therefore, the △*G*_O*_ significant reduced, this implied that TASCs (especially the Fe_2_Ni catalyst) showed a significant performance enhancement toward OER.^[^ ^73^ ^]^ Superb merits of Fe_2_Ni catalyst was also verified by Huang and co‐workers’ work. The authors engineered a series of V‐shaped trinuclear metal–oxygen unit bridged by deprotonated benzenetricarboxylic acid anions (detailed configurations were shown in Figure 5e). As the descriptors of theoretical overpotential, *ȵ* was used to access their trifunctional activity including ORR, OER, and HER, and it was easy to find that Fe_2_Ni catalyst had the most excellent catalytic performance.^[^ ^74^ ^]^

## 4 Modification Engineering in the First Coordination Sphere

### 4.1 Coordination Atoms

As single metal atoms coordinated with other non‐metal atoms, direct regulation on the electronic configuration and geometric structure of these active centers will be observed, which contributes to higher catalytic activity. In this section, we will systematically illustrate atom‐level interface modification engineering focusing on the category, number, and spatial distribution of coordination atoms around central metal sites in SACs.

#### 4.1.1 Coordination with Nitrogen Atoms

Nitrogen atoms that coordinate with metal center in M—N—C catalysts can generally be divided into pyrrolic N, pyridinic N, graphitic N, and oxidized N, and various kinds of nitrogen atoms could have different impact on intrinsic properties of the active center as well as their catalytic performance (summarized in **Table** **3**).

**Table 3:** The oxygen electrocatalysis performance on the catalysts coordinated with N atoms

Catalyst	Active center	Media	Performance (@10 mA cm^−2^)	Reference
SA‐Fe/NG	Fe—N_(po)4_	0.1 m KOH	*E*_1/2_ = 0.88 V	[75]
HP—FeN_4_	Fe—N_(po)4_	0.5 m H_2_SO_4_	*E*_1/2_ = 0.8 V	[76]
Co—N SAC_Dp_	Co—N_(po)4_	0.1 m KOH	FEH2O2 > 70% (>1 V)	[79]
HNC—Co	NH_2_—Co—N_4_	0.5 m H_2_SO_4_	*E*_over_ = 0.265 V	[80]
Fe—N—GDY	Fe—(sp)N_2_C_2_OH	0.1 m KOH	*E*_1/2_ = 0.89 V	[81]

**Figure 6:**
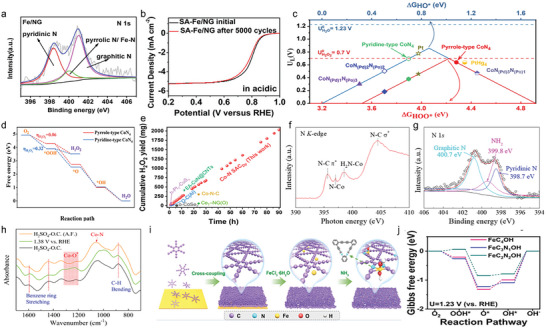
a) High‐resolution N 1s spectrum of SA‐Fe/NG. b) Linear Sweep Voltammetry (LSV) curves of SA‐Fe/NG and before and after 5000 potential cycles in O_2_‐saturated 0.5 m H_2_SO_4_. Reproduced with permission.^[^ ^75^ ^]^ Copyright 2018, The National Academy of Sciences of the United States of America. c) Volcano plot depicting the Gibbs free energy of reaction intermediates (Δ*G*_HO*_ and Δ*G*_HOO*_) on different Co—N coordination structures. d) Free energy diagram of ORR on the pyridine‐type and pyrrole‐type CoN_4_. e) Accumulatively produced H_2_O_2_ for Co—N SAC and previously reported catalysts. Reproduced with permission.^[^ ^79^ ^]^ Copyright 2022, American Chemical Society. f) N K‐edge XANES spectrum. g) XPS spectra of N 1s. h) Operando SR‐FTIR spectroscopy measurements under various potentials for HNC—Co. Reproduced with permission.^[^ ^80^ ^]^ Copyright 2019, American Chemical Society. i) Synthetic process of the Fe—N—GDY. j) Gibbs free energy profiles for ORR on FeC*_x_* N_4−_ *_x_* OH*_y_* (*x* = 2, 3, 4; *y* = 0, 1). Reproduced with permission.^[^ ^81^ ^]^ Copyright 2022, John Wiley and Sons.

Yang et al. synthesized the SA Fe catalysts supported on nitrogen‐doped graphitic carbons (marked as SA‐Fe/NG), and unveiled the role of Fe—pyrrolic‐N species in enhancing the ORR performance under acidic conditions. In **Figure** **6**a, high‐resolution N 1s spectrum of SA‐Fe/NG can be fitted into three peaks, the one located at 400.1 eV corresponded to pyrrolic N whose content (31.9 at%) was apparently higher than that of Fe/NG (11.6 at%). Benefited from the increasing pyrrolic‐N species, SA‐Fe/NG performed outstanding ORR activity and negligible performance degradation after 5000 cycles (Figure 6b). DFT verified that Fe—pyridinic‐N was too weakly bound with oxygen intermediates to be an active site, and intermediates were prone to absorb on C atom adjacent to pyrrolic N. Therefore, Fe—pyrrolic‐N species was regarded as the origin of the improved activity.^[^ ^75^ ^]^ This finding was also supported by the study of Wu and co‐workers. After the pyrolysis in ammonia atmosphere, pyridine‐type Fe—N_4_ sites were transformed into high purified pyrrole‐type Fe—N_4_ species that endowed Fe SAC with improved intrinsic activity, preferable O_2_ adsorption energy and more efficient 4‐electron pathway.^[^ ^76^ ^]^ Owing to the complexity of the M—N—C structure and the influence of other factors on the active center, such as the location of the active site and the tailoring of the electronic structure of metal or nitrogen atoms resulting from additional atoms/ligands, some researchers argued that pyridinic N can also contribute to ORR performance improvement.^[^ ^29^, ^77^, ^78^ ^]^ Hence, there is still some debate about which N species is the real activity source.

Zhu and co‐workers combined both experiments and DFT calculations to identify high active Co—N_4_ coordination for selective oxygen reduction to H_2_O_2_. The 2e^−^ or 4e^−^ ORR pathway is closely related to the formation of HOO* and HO* intermediates during catalytic process. Therefore, △*G*_HOO*_ and △*G*_HO*_ in different coordination environments were simulated. As shown in Figure 6c, pyridine‐type Co—N_4_, Co—N_(pd)3_N_(po)1_, Co—N_(pd)2_N_(po)2_, and Co—N_(pd)1_N_(po)3_ with strong adsorption of HOO* were inclined to break the O—O bond and perform 4e^−^ pathway, while the △*G*_HOO*_ of the pyrrole‐type Co—N_4_ (4.28 eV) was close to the optimal adsorption energy (4.22 eV), allowing pyrrole‐type Co—N_4_ carry out the 2e^−^ reduction process. This was further determined by free energy calculation in Figure 6d, where pyrrole‐type Co—N_4_ was favorable for 2e^−^ ORR pathway and pyridine‐type Co—N_4_ preferred 4e^−^ ORR pathway. This result meant that the protonation of OOH* on pyrrole‐type Co—N_4_ was more favorable than the dissociation kinetics. Electrochemical test results showed that pyrrole‐type Co—N_4_ configuration catalysts displayed a remarkable H_2_O_2_ selectivity of 94% and the H_2_O_2_ yield tested in a flow cell was 2032 mg in 90 h (Figure 6e).^[^ ^79^ ^]^

Apart from the pyrrolic N and pyridinic N, other hetero‐nitrogen have also been reported to have the potential as the catalytic sites for oxygen‐related electrocatalysis. Su et al. designed and prepared hetero‐N‐coordinated H_2_N—Co—N_4_ catalysts by virtue of “imine‐confinement” strategy. In N K‐edge XANES spectrum (Figure 6f), two new peaks located at 396.9 and 398.5 eV were attributed to the excitation of pyridinic N—Co and Co—NH_2_. The assignment of fitted peaks in Figure 6g solidified that the Co center bonded to both pyridinic‐ and amino‐N ligands. EXAFS fitting proved that the coordination number of Co—N and Co—NH_2_ was 4.0 and 1.1, respectively, thus H_2_N—Co—N*_x_* moieties could be determined to be the H_2_N—Co—N_4_ configuration. By using synchrotron radiation‐based Fourier transform infrared spectroscopy (SR‐FTIR), key reaction intermediate O* was observed appearing on Co sites (Figure 6h), which significantly promoted the surface oxo‐species transformation toward efficient OER process.^[^ ^80^ ^]^ Li et al. developed sp‐hybridized N as the anchoring sites of Fe atoms to boost ORR performance. The preparation process and catalyst configuration were shown in Figure 6i, in which graphdiyne (GDY) was an allotrope of carbon that each benzene ring was connected by adiacetylenic linkage (sp‐C). In the process of NH_3_ treatment, N atom replaced C atom in sp‐C to form sp‐N. With more negative charge density, sp‐N manifested the ability to induce more 2p*_x_* + 2p*_y_* electron states approaching to the Fermi level. These electron states could perform strong hybridization with the 3d state of adjacent Fe atoms, resulting in a decrease of 3d state. Therefore, the interaction between Fe center and oxygen intermediate was smoothed and the desorption process of OH* was accelerated because of the electronic effect on Fe—sp‐N sites (Figure 6j).^[^ ^81^ ^]^

#### 4.1.2 Coordination with Doping Heteroatom

In addition to regulating the catalogue of nitrogen, doping other heteroatoms (e.g., O, S, B, P, Cl) into nitrogen‐doped carbon substrates is another general strategy to enhance the catalytic performance of SACs. In this section, we only discuss the particular SACs whose metal atoms coordinate directly with the introduced atoms. These ingenious designs occurring in the first coordination sphere make the catalytic active center asymmetric. While adjusting the electronic structure of metal atoms, the adsorption energy of intermediates in the reaction process could be optimized as well, which is beneficial to further stimulate the catalytic potential of SACs (the performance of these catalysts is summarized in **Table** **4**).

**Table 4:** The oxygen electrocatalysis performance on the catalysts coordinated with heteroatoms

Catalyst	Active center	Media	Performance (@10 mA cm^−2^)	Reference
Ni—O—G	Ni—O_4_	1 m KOH	*E*_over_ = 0.224 V	[82]
Mn/C—NO	Mn—O_1_N_3_	0.1 m KOH	*E*_1/2_ = 0.86 V	[84]
Mn SA@CNSs	Mn—O_2_N_2_	0.1 m KOH	*E*_1/2_ = 0.88 V	[85]
Mn SA@CNSs	Mn—O_2_N_2_	1 m KOH	*E*_over_ = 0.303 V	[85]
S‐4	V—O_4_N_1_	0.1 m KOH	*E*_1/2_ = 0.865 V	[86]
Ni—N_2_O_2_/C	Ni—O_2_N_2_	0.1 m KOH	FE_H2O2_ = 91%@70 mA cm^−2^	[88]
FeN_2_O_2_/HNC	Ni—O_2_N_2_	0.1 m KOH	H_2_O_2_ selectivity > 95% (0.7 V)	[89]
W_1_/NO—C	W—N_1_O_2_	0.1 m KOH	H_2_O_2_ selectivity: 90–98% (0.2–0.7 V)	[90]
ZnO_3_C	Zn—O_3_C	0.1 m KOH	FE_H2O2_ = 90%	[91]
N_4_—Ni_1_—O_2_/OCNTs	Ni—N_4_O_2_	1 m KOH	FE_H2O2_ = 96%@200 mA cm^−2^	[92]
S—Cu—ISA/SNC	Cu—S_1_N_3_	0.1 m KOH	*E*_1/2_ = 0.918 V	[31]
Cu SA/NPSC	Cu—S_1_N_3_	0.1 m KOH	*E*_1/2_ = 0.84 V	[96]
Co_1_—GO	Co—S_2_N_2_	0.1 m KOH	*E*_1/2_ = 0.871 V	[97]
Cu SACs/SNGF	Cu—S_1_O_3_	0.1 m KOH	*E*_1/2_ = 0.862 V	[98]
Pt/HSC	Pt—S_4_	0.1 m HClO_4_	FE_H2O2_ = 96%	[99]
Mo_1_/OSG—H	Mo—S_1_O_3_	0.1 m KOH	FE_H2O2_ > 95%	[100]
S|NiN*_x_*—PC/EG	Ni—S_1_N_3_	1 m KOH	*E*_over_ = 0.28 V	[101]
Mn—NSG	Mn—S_1_N_3_	1 m KOH	*E*_over_ = 0.296 V	[102]
Mo—carbon	Mo—NSO_2_	1 m KOH	*E*_over_ = 0.303 V	[103]
Mo—carbon	Mo—NSO_2_	0.1 m KOH	*E*_1/2_ = 0.788 V	[103]
Fe—NSDC	Fe—S_1_N_3_	1 m KOH	*E*_over_ = 0.41 V	[104]
Fe—NSDC	Fe—S_1_N_3_	0.1 m KOH	*E*_1/2_ = 0.84 V	[104]
Co—N,B—CSs	Co—B_1_N_3_	0.1 m KOH	*E*_1/2_ = 0.83 V	[109]
Zn—B/N—C	Zn—B_1_N_3_	0.1 m KOH	*E*_1/2_ = 0.886 V	[110]
Zn—B/N—C	Zn—B_1_N_3_	0.1 m HClO_4_	*E*_1/2_ = 0.753 V	[110]
Fe—N/P—C‐700	Fe—P_1_N_3_	0.1 m KOH	*E*_1/2_ = 0.867 V	[111]
Fe—N/P—C‐700	Fe—P_1_N_3_	0.1 m HClO_4_	*E*_1/2_ = 0.72 V	[111]
Co—P,N—CNT	Co—P_1_N_3_	0.1 m KOH	*E*_1/2_ = 0.827 V	[112]
Co—P,N—CNT	Co—P_1_N_3_	0.1 m HClO_4_	*E*_1/2_ = 0.8 V	[112]
Ru—Cl—N SAC	Ru—Cl_2_N_2_	0.1 m KOH	*E*_1/2_ = 0.9 V	[113]
Ru—Cl—N SAC	Ru—Cl_2_N_2_	1 m KOH	*E*_over_ = 0.233 V	[113]

##### O Atom Doping

**Figure 7:**
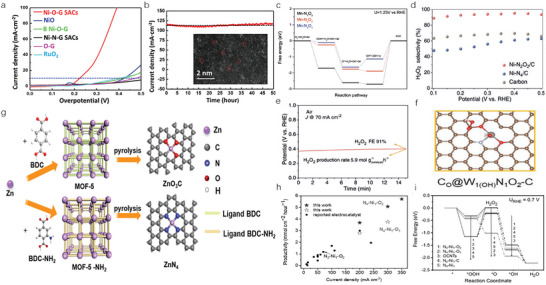
a) The OER current curves of Ni—O—G SACs, NiO, B Ni—O—G, Ni—N—G SACs, O—G, and RuO_2_ tested at 5 mV s^−1^ and 80% *iR* correction in 1 m KOH. b) Chronoamperometric curve of Ni—O—G SACs obtained at constant overpotential of 400 mV in 1 m KOH, with 80% *iR* correction. Reproduced with permission.^[^ ^82^ ^]^ Copyright 2020, John Wiley and Sons. c) Free‐energy diagram of ORR on Mn—N_1_O_3_, Mn—N_2_O_2_, and Mn—N_3_O_1_ surfaces. Reproduced with permission.^[^ ^84^ ^]^ Copyright 2018, John Wiley and Sons. d) H_2_O_2_ selectivity as a function of the applied potential. e) The H_2_O_2_ concentration and the *V*–*t* curve under the condition of entering air in the three‐phase flow cell electrolytic device. Reproduced with permission.^[^ ^88^ ^]^ Copyright 2020, John Wiley and Sons. f) Schematic of the adsorption of OH^−^ on the W atom in W_1_N_1_O_2_—C coordination structure. Reproduced with permission.^[^ ^90^ ^]^ Copyright 2021, John Wiley and Sons. g) Schematic illustration of the preparation process for electrocatalysts ZnO_3_C and ZnN_4_. Reproduced with permission.^[^ ^91^ ^]^ Copyright 2022, John Wiley and Sons. h) H_2_O_2_ production rate of N_4_—Ni_1_—O_2_/OCNTs compared to the reported state‐of‐the‐art electrocatalysts. i) Free energy diagrams for O_2_ reduction on N_4_—Ni_1_—O_2_, N_4_—Ni_1_—O_1_, N_4_—Ni_1_—C, N_4_—Ni_1_, and bare OCNT structures. Reproduced with permission.^[^ ^92^ ^]^ Copyright 2022, John Wiley and Sons.

Compared with the traditional M—N_4_—C configuration, the introduction of oxygen atoms into the first coordination sphere will alter some intrinsic properties of the active center inevitably. For instance, M—O bonds are known to be much weaker than M—N bonds, and this poses a challenge to the protection and stability of M—O in the single‐atom state. Jiang and co‐workers anchored metal nickel in ultrathin graphene‐like carbon sheets where the atomically dispersed nickel sites coordinated with O atoms, successfully preparing the Ni—O_4_—(OH)_2_ catalysts (denoted as Ni—O—G) with novel structure.^[^ ^82^ ^]^ As shown in **Figure** **7**a, ultralow overpotential of 224 mV could be acquired under the current density of 10 mA cm^−2^ in alkaline environment during OER process. It is worth noting that Ni—O—G achieved the distinguished stability without significant degradation for 50 h at exceptionally high current of 115 mA cm^−2^ (Figure 7b). The origin of such impressive performance was investigated by DFT. High oxidation state in single Ni atoms could lower the energy barrier of *O formation, and then resulted in the efficient and durable OER performance for Ni—O—G.

Fe and Co, Mn catalysts are usually regarded as less active for oxygen reduction because of their excessively strong binding with ORR intermediates.^[^ ^5^ ^]^ Inspiration from Mn cofactors in a biosynthetic model where Mn metal centers coordinated with both O and N atoms could perform effective reduction of oxygen into water.^[^ ^83^ ^]^ Yang et al. designed Mn—N_3_O_1_ cofactor to accelerate ORR process. DFT illustrated that O and N atoms regulate Mn d‐electrons to a reasonable state, resulting in proper electron interactions with oxygen‐bearing intermediates (Figure 7c). This was conducive to intermediates’ adsorption and desorption.^[^ ^84^ ^]^ Mn—N_2_O_2_ catalysts had been engineered by Zhao and co‐workers. Optimized coordination arrangements in Mn—N_2_O_2_ catalysts tuned the adsorption strength of intermediates on Mn site and endowed catalysts with excellent catalytic performance (*E*_1/2_ = 0.88 V in 0.1 m KOH solution) and power density (177 mW cm^−2^) in Zn–air battery.^[^ ^85^ ^]^ Similar to Mn—N_3_O_1_ cofactor, the design of V—O_4_N_1_ took examples from nature. DFT and projected density of states (PDOS) indicated that V—O_4_N_1_ possessed the best configuration and highest density of states at the Fermi level among all kinds of V catalysts with V—O coordination (V—O_5_, V—N_1_O_4_, V—N_2_O_3_, V—N_3_O_2_, V—N_4_O_1_).^[^ ^86^ ^]^

In the attempt to develop metal SACs for 2e^−^ ORR pathway, inspiration can be found in homogeneous catalysis where metal–Schiff‐based catalysts could selectively reduce O_2_ to H_2_O_2_ at low overpotentials.^[^ ^35^, ^87^ ^]^ Such metal–Schiff‐based catalysts typically contain metal centers coordinated with double O atoms and double N atoms (tetradentate M—N_2_O_2_ structure). Zhang and co‐workers chose nickel as the active center and prepared Ni—N_2_O_2_ catalysts with high selectivity and yield toward H_2_O_2_ production. In 0.1 m KOH electrolyte, they used rotating ring disk electrode to perform the selectivity of H_2_O_2_ production, and a maximum of about 96% at potentials between 0.4 and 0.5 V was got (Figure 7d). Moreover, the stability was tested in an electrolytic device under the current density of 70 mA cm^−2^, and Ni—N_2_O_2_ catalysts maintained high Faradaic efficiency of 91% over 8 h (Figure 7e), demonstrating the robust durability under high current conditions.^[^ ^88^ ^]^ With the introduction of O atoms, the d‐band of Fe—N_2_O_2_ catalysts shifted down, thus weakening the adsorption of intermediates and promoting the 2e^−^ pathway.^[^ ^89^ ^]^

In addition to above SACs with M—N_2_O_2_ structure, other O‐doping coordination configuration catalysts can also be used for producing H_2_O_2_. For instance, W—N_1_O_2_ catalysts having the special three serrated coordination structure achieved outstanding selectivity (>90% from 0.2 to 0.7 V) of H_2_O_2_ production in 0.1 m KOH. Theoretical calculation showed that the C atom near O was the active site (Figure 7f) with the best adsorption energy, which was convenient for the adsorption and desorption of HOO*.^[^ ^90^ ^]^ Li and co‐workers reported two kinds of MOF‐based ORR electrocatalysts executing different reaction paths. They were synthesized via regulating the type of ligand (Figure 7g). Zn—N_4_ was obtained by the pyrolysis of MOF‐5—NH_2_ precursor and tended to produce H_2_O through the 4e^−^ pathway, whereas the Zn—O_3_C catalysts generated by sintering MOF‐5 precursor displayed excellent Faradaic efficiency (FE, 90%) of H_2_O_2_ production. The reaction pathway of above two catalysts was determined by the difference of coordination environment. Oxygen is much more electronegative than nitrogen, thus it trapped more electrons from Zn. The remodulation of the electronic structure reduced the d‐band of Zn in Zn—O_3_C, allowing the HOO* intermediate adsorbed on active sites for preferential hydrogenation toward H_2_O_2_ generation.^[^ ^91^ ^]^ Xiao et al. chose multiwalled carbon nanotubes as substrates and introduced two additional O atoms in conventional M—N_4_ coordination (donated as Ni/OCNTs). This six‐coordination catalyst was preferable for 2e^−^ ORR to generate H_2_O_2_, achieving >90% FE_H2O2_ under current densities of 300 mA cm^−2^ and maintaining high FE of ≈96% at 200 mA cm^−2^ under 24 h continuous operation. What impressed the most was that the H_2_O_2_ productivity of Ni—N_4_—O_2_ at 350 mA cm^−2^ was 5.7 mmol cm^−2^ h^−1^, surpassing the best performance reported so far (Figure 7h). It is well‐known that the selectivity of H_2_O_2_ was determined by the desorption energy barrier of HOO* and the dissociation energy barrier of HOO* into O* and OH^−^. The free energy for desorption (0.31 eV) was lower than dissociation (0.33 eV) in Ni—N_4_—O_2_, thus the 2e^−^ pathway was much more preferred for Ni—N_4_—O_2_ (Figure 7i).^[^ ^92^ ^]^

##### S Atom Doping

As a member of the same main group with oxygen, sulfur has gained more and more attention in the atomic‐level interface regulation engineering. According to the past reports, S atoms could act as environmental atoms, which are doped in the carbon skeleton and modify the active center through a long range delocalization effect.^[^ ^78^, ^93^, ^94^, ^95^ ^]^ However, when sulfur atoms enter into the first coordination sphere and coordinate with the metal center directly, novel reaction mechanisms will be expected.

**Figure 8:**
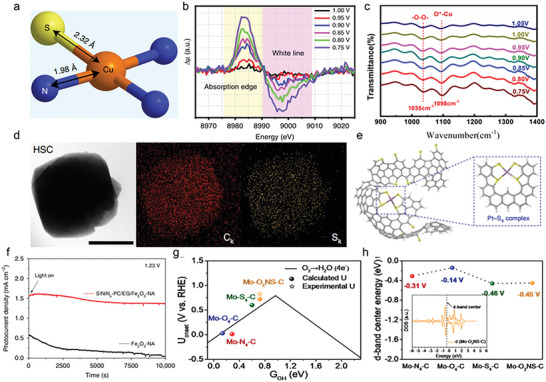
a) Schematic atomic interface model of S—Cu—ISA/SNC. b) Differential Δ*µ* XANES spectra obtained by subtracting the normalized spectrum at every potential to the spectrum recorded at 1.05 V versus RHE. c) Electrochemical in situ FTIR spectra at different potentials of the S—Cu—ISA/SNC. Reproduced with permission.^[^ ^31^ ^]^ Copyright 2020, Springer Nature. d) Energy dispersive X‐ray mapping images of C (red) and S (yellow). e) Proposed atomistic structure of the Pt/HSC. Reproduced with permission.^[^ ^99^ ^]^ Copyright 2016, Springer Nature. f) Transient photocurrent responses of Fe_2_O_3_—NA and S|NiN*_x_*—PC/EG/Fe_2_O_3_—NA (PC and EG correspond to porous carbon and exfoliated graphene, respectively) under AM 1.5G irradiation at 1.23 V. Reproduced with permission.^[^ ^101^ ^]^ Copyright 2019, Springer Nature. g) ORR volcano activity plot. The computed and experimental onset potentials are plotted versus the chosen reaction descriptor *G*_OH_. h) Comparison of d‐band centers for Mo—N_4_—C, Mo—O_4_—C, Mo—S_4_—C, and Mo—O_2_NS—C. Reproduced with permission.^[^ ^103^ ^]^ Copyright 2022, Elsevier.

Shang et al. proposed a strategy to enhance ORR performance by partially replacing coordinated nitrogen atoms with sulfur atoms around Cu active centers (the catalyst was donated as S‐Cu‐ISA/SNC). This unsymmetrical coordination structure was demonstrated by XANES and EXAFS collectively, and its atom interface was shown in **Figure** **8**a. In order to monitor the dynamic evolution of active sites, in situ XANES spectra were employed for examining the variation of atomic and electronic structure around Cu centers during the action process. From 1.05 to 0.75 V, the edge position of Cu K‐edge moved to the lower energy and the density of the white line decreased (Figure 8b), indicating that the valence of Cu species reduced from ≈+2 to +1. Furthermore, the Cu—N peaks appeared as an obvious low‐*R* move from 1.55 to 1.49 Å on the basis of in situ EXAFS results that meant the local structure of the Cu—S_1_N_3_ was changed. The variation of Cu—N bond length was detected by ex situ spectroscopy, shortening from 1.98 to 1.94 Å (0.90 V) and 1.93 Å (0.75 V) under working conditions. Therefore, the most possible geometric configuration was considered as an isolated unsymmetrical Cu—S_1_N_3_ moiety linked with the OOH*, O*, and OH*. The adsorption of these intermediates was also observed by in situ FTIR (Figure 8c). All the above in situ spectroscopy analyses revealed that the low valence (+1) Cu—N‐bond‐shrinking HOO—Cu—S_1_N_3_, O—Cu—S_1_N_3_, and HO—Cu—S_1_N_3_ species might contribute to the enhanced ORR activity.^[^ ^31^ ^]^ Chen et al. also engineered Cu—S_1_N_3_ catalyst with unsymmetrical sulfur coordination, resulting in superior electrochemical performance with a high open‐circuit voltage (1.41 V) and a large power density (138.2 mW cm^−2^) in Zn–air battery.^[^ ^96^ ^]^ The SAC with Co—N_2_S_2_ configuration was attempted via Su and co‐workers. On account of the sulfur atom having a larger radius, the introduction of double sulfur atoms broke the square‐planar coordination structure. Thus, Co—N_2_S_2_ sites existed outside the graphene plane, resulting in a different spatial distribution of Co electrons. N and S atoms realized the optimization of Co electron density and spatial configuration together.^[^ ^97^ ^]^ Making good use of DFT, Xu et al. explored the mechanism by which ORR activity of Cu—O_3_S_1_ was superior to that of Cu—O_4_. Compared with Cu—O_4_, stronger orbital overlaps were observed between valence p orbitals of dopant site (O_3_S) and 3d orbital of Cu, thus leading to relatively weaker *OH interaction with Cu—O_3_S_1_ and promoting the *OH desorption.^[^ ^98^ ^]^

In 2016, Choi and co‐workers reported a sulfur‐doped zeolite‐templated carbon catalyst (donated as Pt/HSC) where platinum could be stabilized in the form of atomically dispersed species. Energy dispersive X‐ray mapping images in Figure 8d indicated the uniform dispersion of S, and the content was up to 17 wt%. As shown in Figure 8e, Pt with four sulfur atoms formed a highly coordinated planar quadrate structure. When Pt—S_4_ was dissolved in water, the platinum ion can easily react with two water molecules and lost double sulfur atoms, causing the distortion of the platinum center and a series of electron transfers to accelerate the reaction process.^[^ ^99^ ^]^ The introduction of S has the potential to change the adsorption behavior of active metal sites toward efficient 2e^−^ ORR. In the catalysts of Mo—O_3_S_1_, the critical OOH* adsorption is significantly enhanced compared with pure Mo—O_4_ catalysts.^[^ ^100^ ^]^

Hou et al. fabricated Ni—S_1_N_3_ through pyrolyzing a mixture of Ni salt and small organic molecules as the N and S sources. The substitution of a nitrogen atom by a sulfur atom resulted in the shortening of the Ni—N bond distance as well as the local distortion of the active site structure, which optimized the electron density of states around the metal center and enhanced the electron transfer ability of catalysts. Ni—S_1_N_3_ was also integrated into Fe_2_O_3_ for solar water oxidation, achieving an AM 1.5G photocurrent density of 1.58 mA cm^−2^ at 1.23 V that was superior to other Fe_2_O_3_—NA) inorganic photoanodes (Figure 8f).^[^ ^101^ ^]^ Recently, Bai et al. explained that, after S introduction, the electron donating ability of Mn atom was reduced in Mn—S_1_N_3_, and thus the formation of O—O bond was expedited, which was the rate‐determining step for this series of SACs.^[^ ^102^ ^]^

Sulfur‐doped catalysts with bifunctional activity have also been widely reported. In a recent study by Zhao et al., Mo—O_2_NS offered better bifunctional catalysis activity than Mo—N_4_ and Mo—O_4_ catalysts because their extreme strong adsorption with O* restricted the production of OH*. Whereas in the Mo—O_2_NS, the downward shift of d‐band center led to the decrease of antibonding state, thus resulting in the weakening of adsorption of O* (Figure 8g,h).^[^ ^103^ ^]^ Zhu and co‐workers developed Fe—S_1_N_3_ with excellent bifunctional activity. The half‐wave potential during ORR and overpotential during OER in alkaline environment were 0.84 V and 410 mV, respectively.^[^ ^104^ ^]^

##### B/P/Cl Atom Doping

Since the doping of S and O atoms has improved the performance of SACs successfully, it is easy to think that the introduction of other nonmetallic elements may have the potential for tailoring the electronic structure of metal center. This has been confirmed by many theoretical calculation researches.^[^ ^105^, ^106^, ^107^, ^108^ ^]^

**Figure 9:**
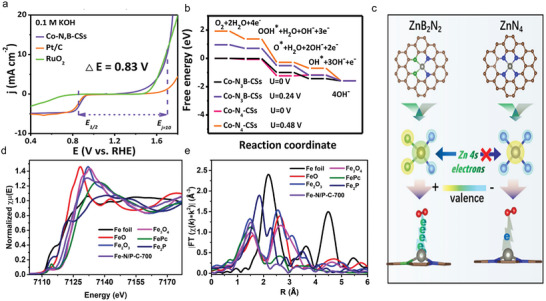
a) LSV curves of different catalysts for both ORR and OER in 0.1 m KOH at 1600 rpm and a sweep rate of 5 mV s^−1^. b) Free‐energy paths of ORR on Co—N_3_B—CS and Co—N_4_—CS systems during the ORR in alkaline solution at the equilibrium potential of *U* = 0 and 0.24 V for Co—N_3_B—CSs and *U* = 0, 0.48 V for Co—N_4_—CSs. Reproduced with permission.^[^ ^109^ ^]^ Copyright 2018, American Chemical Society. c) Diagram for facile charge transfer of ZnB_2_N_2_ compared with ZnN_4_ moiety. Reproduced with permission.^[^ ^110^ ^]^ Copyright 2021, John Wiley and Sons. d) Comparison of Fe K‐edge XANES spectra of Fe—N/P—C‐700 and control samples. e) EXAFS spectra of Fe—N/P—C‐700 and control samples. Reproduced with permission.^[^ ^111^ ^]^ Copyright 2020, American Chemical Society.

Mu and co‐workers constructed asymmetrically coordinated Co—B_1_N_3_ catalysts by pyrolysis and acid treatment. The introduction of B atom enhanced the unbalanced charge distribution of carbon substrate and endowed Co—B_1_N_3_ with Pt/C‐like bifunctional oxygen electrocatalytic activity. It could be found that the Δ*E* (a descriptor that describes dual function electrocatalytic performance) was 0.83 V in **Figure** **9**a. At *U* = 0.24 V, DFT illustrated that the free energy of each step showed a downward trend (Figure 9b).^[^ ^109^ ^]^ With the configuration of M—B_2_N_2_, Zn—B_2_N_2_ performed general ORR activity over a wide pH range. The half‐wave potential in 0.1 m KOH and 0.1 m HClO_4_ were 0.886 and 0.753 V, respectively. Compared with Zn—N_4_, Zn in the Zn—B_2_N_2_ had more electron density in its 4s orbit. Owing to the localization of 4s electrons, Zn—B_2_N_2_ had a more suitable adsorption ability for oxygen (Figure 9c).^[^ ^110^ ^]^

Yuan et al. used the differences in charge polarization, electron spin density, and electronegativity between C and P atoms to design Fe—P_1_N_3_ catalysts for promoting O adsorption and O—O bond fracture. XPS results indicated that atomically dispersed Fe possessed typical Fe—N and Fe—P dual‐coordinating environments, and that was further confirmed by XANES (Figure 9d) and EXAFS (Figure 9e). The coordination numbers of N and P atoms were calculated to be 3.1 ± 0.3 and 0.9 ± 0.1, respectively. Fe—P_1_N_3_ catalysts displayed all‐pH ORR applicability.^[^ ^111^ ^]^ In Co—P_1_N_3_ catalyst, general 4e^−^ ORR activity in acidic and alkaline environments could also performed.^[^ ^112^ ^]^

A novel Ru single atom catalyst coordinated with Cl and N atoms was fabricated via hydrothermal method. Ru—Cl—N SACs, with well‐defined Ru—Cl_2_N_2_ structure, served as multifunctional electrocatalysts, which had superior half‐wave potential of 0.9 V for ORR and the ultralow overpotential of 233 mV to deliver a current density of 10 mA cm^−2^ during OER. In order to verify the practical application of the catalysts, Ru—Cl—N was assembled as cathode in Zn–air battery, and its specific capacity could achieve 804.26 mAh g^−1^ without significant decay within 360 h.^[^ ^113^ ^]^

### 4.2 Planar Coordination Number

Among the most classical M—N—C structural catalysts, the planar tetra‐coordinated structure (linked to four nitrogen atoms) tends to be the most common. However, M—N_4_ catalysts do not always achieve optimal adsorption of intermediates. As demonstrated by recent studies, reducing the coordination number of nitrogen is a viable strategy to improve the performance of SACs.^[^ ^114^, ^115^ ^]^ In this section, we will discuss the influence of coordination number in planar configuration, and three types of catalysts with M—N_2_, M—N_3_, and M—N*_x_* C_4−_ *_x_* structures will be introduced as examples (the performance of M—N*_x_* catalysts is summarized in **Table** **5**).

**Table 5:** The oxygen electrocatalysis performance on the M—N*_x_* catalysts

Catalyst	Active center	Media	Performance (@10 mA cm^−2^)	Reference
Cu—NC‐60	Cu—N_2_	0.1 m KOH	*E*_1/2_ > 0.8 V	[117]
Co—C_3_N_4_	Cu—N_2_	1 m KOH	*E*_over_ = 0.31 V	[118]
Fe—N—C‐900	Fe—N_2_	0.1 m KOH	*E*_1/2_ = 0.927 V	[119]
FeN_2_/NOMC‐3	Fe—N_2_	0.1 m KOH	*E*_1/2_ = 0.863 V	[120]
ZnNC	Zn—N_2_	0.1 m KOH	*E*_1/2_ = 0.857 V	[121]
Cu—NGS	Cu—N_2_	0.1 m KOH	*E*_1/2_ = 0.81 V	[122]
Co—N_2_—C/HO	Co—N_2_	0.1 m KOH	H_2_O_2_ selectivity: 96%	[123]
CUMSs—ZIF‐67	Co—N_3_	1 m KOH	*E*_over_ = 0.32 V	[124]
CUMSs—ZIF‐67	Co—N_3_	0.5 m KBi	*E*_over_ = 0.41 V	[124]
Cu SAs/NC‐900	Cu—N_3_	0.1 m KOH	*E*_1/2_ = 0.87 V	[125]
Co—N_3_C_1_@GC	Co—N_3_C_1_	0.1 m KOH	*E*_1/2_ = 0.824 V	[126]
Zn—N_3_C—C_8_	Zn—N_3_C_1_	0.1 m KOH	*E*_1/2_ = 0.91 V	[127]
Co—N_3_—C	Co—N_3_C_1_	0.1 m KOH	*E*_1/2_ = 0.891 V	[128]
Mn SAC	Mn—N_2_C_2_	0.1 m KOH	*E*_1/2_ = 0.915 V	[129]
Mn SAC	Mn—N_2_C_2_	0.1 m KOH	*E*_over_ = 0.35 V	[129]
Cu SA/NC	Cu—N_2_C_2_	0.1 m KOH	*E*_1/2_ = 0.898 V	[130]
Cu/CNT‐8	Cu—N_2_C_2_	0.1 m KOH	*E*_1/2_ = 0.863 V	[131]
Ni SA@NCA	Ni—N_2_C_2_	1 m KOH	*E*_over_ = 0.43 V	[132]
Pt_1_—C_2_N_2_ SAC	Pt—N_2_C_2_	0.5 m H_2_SO_4_	*E*_over_ = 0.232 V	[133]
Pt_1_—C_2_N_2_ SAC	Pt—N_2_C_2_	0.5 m H_2_SO_4_	*E*_over_ = 0.405 V@120 mA cm^−2^	[133]

#### 4.2.1 M—N_2_ Moieties

**Figure 10:**
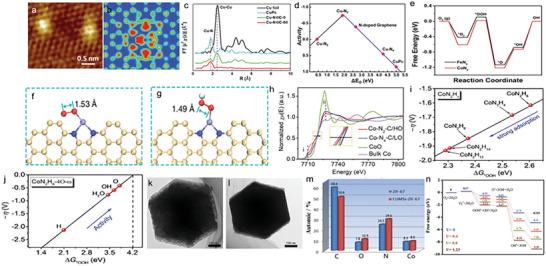
a) STM, and b) STM simulation images of Cu—NC‐60. c) EXAFS spectra of the Cu K‐edge. d) Volcano plot of the relationship between ORR activity and ∆*E*_O_. Reproduced with permission.^[^ ^117^ ^]^ Copyright 2016, The Royal Society of Chemistry. e) Free energy diagram of the ORR on a FeN_2_ and on CoN_2_ sites. Reproduced with permission.^[^ ^119^ ^]^ Copyright 2018, John Wiley and Sons. f) O_2_ and g) OOH adsorption configurations on Zn—N_2_ active site. Reproduced with permission.^[^ ^121^ ^]^ Copyright 2019, Springer Nature. h) Co K‐edge XANES spectra. i) The plot of *η* as a function of ∆G_*OOH_ for the CoN_2_H*_x_* (*x* = 4, 6, 8, 12, 14) moieties. j) The introduced solvent species on the Co site of CoN_2_H_6_ with 4 epoxy groups. Reproduced with permission.^[^ ^123^ ^]^ Copyright 2021, John Wiley and Sons. TEM images of k) ZIF‐67 and l) CUMS—ZIF‐67. m) The element content in ZIF‐67 and CUMS—ZIF‐67. Reproduced with permission.^[^ ^124^ ^]^ Copyright 2017, Elsevier. n) Free energy diagrams for ORR process on CuN_3_ at different overpotentials. Reproduced with permission.^[^ ^125^ ^]^ Copyright 2020, John Wiley and Sons.

During operation, Cu(II)—N generally needs to be converted to Cu(I)—N for binding and activation of O_2_ by applying the potential.^[^ ^116^ ^]^ Therefore, Wang and co‐workers used CuPc and dicyandiamide as the coprecursors, achieving a high density of Cu(I)—N sites embedded in graphene. It is worth mentioning that the loading of Cu in Cu(I)—N catalysts (denoted as Cu‐NC‐60) reached 8.5 wt%, surpassing the conventional loading of SACs (<1.5 wt%). **Figure** **10**a displayed a scanning tunneling microscope (STM) image where the bright dot was attributed to the copper center and its neighboring C and N atoms. The STM simulation (Figure 10b) suggested that a Cu—N_2_ center was embedded in the graphene lattice, in accordance with STM topography. This conclusion was further demonstrated by EXAFS spectroscopy (Figure 10c). The characteristic peak at 1.9 Å corresponded to the Cu—N bond, and there is no peak at 2.5 Å (Cu—Cu bond), implying that the Cu atoms were only bonded with N atoms in Cu‐NC‐60. EXAFS fitting results showed that the coordination number of Cu was about 2.2, which strongly proved the configuration of Cu—N_2_ active site. In 0.1 m KOH solution, the ORR performance of Cu‐NC‐60 was superior to Ag catalysts and 40 wt% Pt/C catalysts. The better activity was explored by theoretical calculations (Figure 10d). Compared with Cu—N_3_/N_4_, CuPc, and N‐doped graphene, Cu‐NC‐60 exhibited the optimal binding strength of O species, therefore, possessed the best adsorption/desorption capacity for key intermediates.^[^ ^117^ ^]^ Qiao and co‐workers also discovered that the lower coordination number contributed to the high catalytic activity by combining both experiments and DFT calculations. The Co—N_2_ active site loaded on g‐C_3_N_4_ proved to have promising potential for ORR and OER bifunctional catalysis.^[^ ^118^ ^]^ Zhu et al. designed M—N—C (M = Co and Fe) SACs with robust M—N_2_ active moieties enabled the enhanced ORR performance. The atom interface configurations of Fe—N_2_ and Co—N_2_ were confirmed by XANES and EXAFS. In contrast to Co—N_2_, the barrier for releasing OH* intermediate in Fe—N_2_ was even lower (Figure 10e). Therefore, Fe—N_2_ was more favorable for the oxygen reduction process.^[^ ^119^ ^]^ A similar mechanism was also found by Guo and co‐workers, who found that the ORR performance of Fe—N_2_ was better than that of Fe—N_4_ due to its more appropriate interactions with OH* and O_2_*.^[^ ^120^ ^]^ Li et al. identified the structural activation of Zn—N_2_ active site. Different from the normal bond length of O—O (1.21 Å), the O—O bond of OOH* and O_2_* adsorbed on Zn—N_2_ sites was significantly enhanced (Figure 10f,g). The high degree of O—O bond stretching can accelerate the 4e^−^ oxygen reduction of Zn—N_2_.^[^ ^121^ ^]^ Using the reductive property of thiourea promoted Cu—N bond breakage and Cu^II^—N_4_ to Cu^I^—N_2_ conversion during pyrolysis, Wang et al. designed and synthesized Cu—N_2_ catalysts whose ORR performance (*E*_1/2_ = 0.81 V) was comparable to that of commercial Pt/C (*E*_1/2_ = 0.83 V). DFT illustrated that Cu—N_2_ could lower the free energy barrier at each step during the reaction.^[^ ^122^ ^]^

For the selective generation of H_2_O_2_, it is necessary to ensure the appropriate binding energy between the active site and the OOH* intermediate, so that it could retain the O—O bond, rather than dissociation of O—O into O* and OH*. In order to achieve this goal, Gong et al. prepared Co—N—C catalysts rich in epoxy groups, and obtained high selectivity (91.3%) toward H_2_O_2_ production and excellent quality activity (44.4 A g^−1^ at 0.65 V). As shown in Figure 10h, the characterization of active sites was performed by XAFS. Compared with Co—N_4_, the ii peak transition in the Co K‐edge of Co—N—C significantly reduced, indicating that there are defective graphene structures and low coordination environment around Co. According to the fitting results of EXAFS, the coordination number of Co—N—C is 2.0. Thus, the active site was identified as Co—N_2_ structure. To investigate the effect of oxygen functional groups, the authors simulated the Gibbs free energy of OOH* for different coordination environments. First, a serial of Co—N_2_H*_x_* moieties (Co—N_2_H*_x_*, *x* = 4, 6, 8, 12, 14) were constructed. Figure 10i showed the plot for ORR activity in terms of *η* as a function of ∆G_*OOH_, illustrating that all considered moieties have too strong adsorption of the *OOH intermediate for the rate‐determining step (*OOH + e^−^ + H^+^ → H_2_O_2_). Whereas in the coordination environment with oxygenated species groups (O, H_2_O, and OH), the OH and O solvent species were the most effective in weakening the adsorption strength of *OOH on the Co site, leading to the significantly enhanced catalytic activity (Figure 10j). The above evidence indicated that the epoxy group and the Co—N_2_ sites synergistically enhanced the 2e^−^ ORR performance.^[^ ^123^ ^]^

#### 4.2.2 M—N_3_ Moieties

It is known that the Co ion in ZIF‐67 is coordinated with four strong imidazole ligands, and this saturated coordination structure is not conducive to the adsorption of intermediates. In order to create accessible adsorption sites, Wang and co‐workers prepared coordinately unsaturated metal site (CUMS)‐enriched ZIF‐67 treated by plasma radiation. TEM images showed that CUMS—ZIF‐67 (Figure 10k) became slightly transparent and droopy compared with ZIF‐67 (Figure 10l), illustrating that the atomic coordination geometry had been modified during the plasma radiation treatment. XPS revealed the variation in the coordination structure. Specifically, the atomic ratio of C/N went from 0.2 to 0.56 pointing to the geometry destruction in CUMS—ZIF‐67 (Figure 10m). Moreover, the proportion of Co—N*_x_* had increased from 6.55% to 13.45%, indicating that the ligand was removed from Co—N_4_ configuration after plasma irradiation, and the open Co site was generated in the process. Then, the authors used XANES and EXAFS to verify the coordination structure of Co—N*_x_* as Co—N_3_. In order to explore the role of CUMSs, the ligands in Co—N_3_ were reconstructed, and then there was a significant decrease in OER performance. Interestingly, the activity of reconstructed ZIF‐67 was restored when the plasma radiation treatment was repeated, proving that CUMS was the real source of the performance improvement.^[^ ^124^ ^]^ By controlling the annealing conditions (900 °C), Ma et al. prepared unsaturated Cu—nitrogen architecture (Cu—N_3_ moieties) catalysts. Benefitting from the adjustment of coordination structure, Cu—N_3_ displayed a higher half‐wave potential of 870 mV and 10 times turnover frequency than that of CuN_4_. Theoretical calculation in Figure 10n showed that the low coordination number contributed to the formation of O_2_* intermediate.^[^ ^125^ ^]^

#### 4.2.3 M—N_x_C_4−x_ Moieties

**Figure 11:**
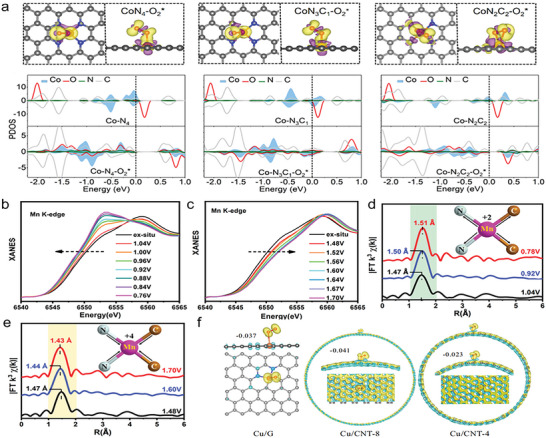
a) Calculated charge density distributions, and projected density of states (PDOS) for the Co center, atoms in the first coordinate shell, and oxygen of Co—N_4_, Co—N_3_C_1_, and Co—N_2_C_2_, respectively. Reproduced with permission.^[^ ^126^ ^]^ Copyright 2020, American Chemical Society. Operando XAFS characterization of Mn SAC. Mn K‐edge XANES spectra during b) ORR and c) OER. EXAFS spectra during d) ORR and e) OER. Reproduced with permission.^[^ ^129^ ^]^ Copyright 2020, American Chemical Society. f) Side view and top view of the charge density difference for three models with O_2_*. Reproduced with permission.^[^ ^131^ ^]^ Copyright 2021, Springer Nature.

For the classical tetra‐coordination structure, when some nitrogen atoms are replaced by carbon atoms, the electronic structure and band distribution of the metal center will change accordingly. Lu and co‐workers discovered that the N/C atom ratio had a significant effect on the distribution of Co d states near Fermi level. Compared with Co—N_2_C_2_ and Co—N_4_, Co—N_3_C_1_ had an obvious overlap between the DOS of Co d and the empty O_2_ 2π* states, which was conducive to the electron hybridization of O_2_ and the protonation process for the adsorption of O_2_* (**Figure** **11**a).^[^ ^126^ ^]^ Zhang et al. constructed atomically dispersed Zn on ultrathin 2D N‐doped carbon nanosheets with Zn—N_3_C—C_8_ sites. This structure exhibited near‐Fermi electronic states that differ from the graphene‐enclosed Zn—N_4_—C_10_ sites and divacancy trans‐Zn—N_2_C_2_—C_8_ sites, enabling more active site exposure and faster electron transport.^[^ ^127^ ^]^ Atomic level interface modification also has the ability to optimize the reaction path. In the research of Wu and co‐workers, Co—N_3_C_1_ was reported to bind with hydroxyl groups during the reaction, and followed the reaction pathway: Co—N_3_C_1_ + 2O_2_ + 7e^−^ + 4H_2_O → Co—N_3_C_1_—OH + 7OH^−^. This is different from the pathway of Co—N_4_: Co—N_4_ + O_2_ + 4e^−^ + 2H_2_O → Co—N_4_ + 4OH^−^. Optimized reaction path endowed Co—N_3_C_1_ with faster reaction kinetics, thus the △*G* of rate‐determining step was significantly reduced and superb ORR activity was got.^[^ ^128^ ^]^

The coordination environment of the M—N_2_C_2_ structure has also triggered extensive research interest. Shang et al. tracked the changes of Mn K‐edge absorption spectra during ORR and OER to identify the dynamic evolution of active sites. The characterizations of Mn—N_2_C_2_ were performed in ORR (1.04–0.78 V vs RHE) and OER (1.48–1.70 V vs RHE) potential windows shown in Figure 11b,c, and valence state of Mn was observed to reduce from 2.9 to 2.2 (from ≈+3 to +2) in ORR process and changed from 3.1 to 3.8 (from ≈+3 to +4) during OER. In situ EXAFS revealed the variation of geometric construction. The peak position of Mn—N and Mn—C in Mn—N_2_C_2_ (Figure 11d,e at 0.78 and 1.7 V) displayed different skewing, indicating the extending or shrinking of bond length. Therefore, Mn^2+^—N_2_C_2_ with prolonged bond length was considered to be the active site during ORR, while the Cu—N‐bond‐shrinking Mn^4+^—N_2_C_2_ was reckoned to the active site during OER.^[^ ^129^ ^]^ Sun et al. adjusted the ratio of Cu^1+^ SA (Cu—N_2_C_2_) and Cu^2+^ SA (Cu—N_4_) by changing the content of urea in the precursor in virtue of the reductive nature of urea during pyrolysis. Excellent performance was due to the advantages of atomically dispersed Cu^1+^ sites and mesoporous structures in enhancing material transport.^[^ ^130^ ^]^ Du and co‐workers anchored Cu—N_2_C_2_ sites on the 8 nm CNT, and modulated the active site geometric distortion and ORR activity via novel substrate strain strategy. Their research demonstrated that reasonable distortions could strengthen the Cu—O bond as well as facilitate the transfer of electrons from the Cu to nearby O atoms (charge density distribution shown in Figure 11f).^[^ ^131^ ^]^ Ni—N_2_C_2_ catalyst confirmed that there was an reinforced density of states near the Fermi level, which was conducive to the formation of O* in the rate‐determining step.^[^ ^132^ ^]^ Liu and co‐workers reported a Pt‐based catalyst with unique Pt—N_2_C_2_ structure for OER under acidic conditions. High‐valence Pt^(2.4+^ *^*δ*^* ^)+^ active center manifested low *E*_over_ of 405 mV at 120 mA cm^−2^ in 0.5 m H_2_SO_4_ even under 12 h operation. It is mentioned that key (*O)—Pt—C_2_N_2_ intermediate was observed via in situ synchrotron radiation infrared spectroscopies during the reaction, which is helpful for accelerating the dissociation of H_2_O.^[^ ^133^ ^]^

### 4.3 Axial Coordination Configuration

As mentioned above, the modification strategies, including heteroatom doping method and coordination number adjustment approach, seek to break the square planar symmetry of M—N_4_ sites, thus reducing the adsorption energy of oxygen‐related intermediates during ORR/OER. As demonstrated by recent studies, when the fifth (or higher) atom coordinated with the metal center from the axial direction, the axial charge of the metal atom could be redistributed, which was beneficial to regulate the binding strength between the active site and oxygen species involving orbital overlap in the axial direction with respect to the M—N_4_ plane.^[^ ^134^, ^135^ ^]^ The design of axial coordination provides a novel perspective for atom‐level interface modification engineering and is expected to help us discover new catalytic mechanisms (the oxygen electrocatalysis performance on the catalysts coordinated with axial atoms is summarized in **Table** **6**).

**Table 6:** The oxygen electrocatalysis performance on the catalysts coordinated with axial atoms

Catalyst	Active center	Media	Performance (@10 mA cm^−2^)	Reference
PFePc—I	Fe—N_4_—I	0.1 m KOH	*E*_1/2_ = 0.948 V	[134]
FeN_4_—O—NCR	Fe—N_4_—O	0.1 m KOH	*E*_1/2_ = 0.942 V	[136]
O—Zr—N—C	Zr—N_4_—O	0.1 m KOH	*E*_1/2_ = 0.91 V	[137]
Fe(Zn)—N—C	O—N_4_Fe—O—FeN_4_—O	0.1 m HClO_4_	*E*_1/2_ = 0.83 V	[138]
Fe—N—C	Fe—N_4_—O_2_	0.1 m HClO_4_	*E*_1/2_ = 0.81 V	[139]
Fe—N—C	Fe—N_4_—O_2_	0.1 m KOH	*E*_1/2_ = 0.9 V	[139]
FeCl_1_N_4_/CNS	Fe—N_4_—Cl	0.1 m KOH	*E*_1/2_ = 0.921 V	[140]
FeN_4_Cl_1_/NC	Fe—N_4_—Cl	0.1 m KOH	*E*_1/2_ = 0.91 V	[141]
FeN_4_Cl_1_/NC	Fe—N_4_—Cl	0.1 m HClO_4_	*E*_1/2_ = 0.79 V	[141]
Fe—N/C SAC	Fe—N_4_—Cl	0.1 m KOH	*E*_1/2_ = 0.91 V	[142]
WN_5_	W—N_5_	0.1 m KOH	*E*_1/2_ = 0.88 V	[144]
WN_5_	W—N_5_	0.1 m HClO_4_	*E*_1/2_ = 0.77 V	[144]
Fe SAC/N—C	Fe—N_5_	0.1 m KOH	*E*_1/2_ = 0.89 V	[145]
Ni MOF NSs‐6	Ni—N_5_ (approximately)	0.1 m KOH	H_2_O_2_ selectivity: 98%	[146]

#### 4.3.1 Axial Coordination with Heteroatoms

**Figure 12:**
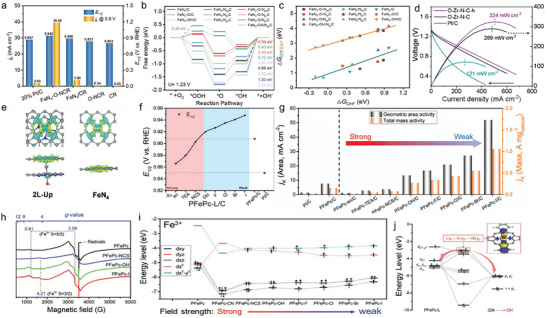
a) Comparison of *E*_1/2_ and *j*_k_ at 0.9 V. b) Free energy diagram for ORR on various FeN_4_‐based models. c) Scaling relationships between the adsorption free energies. Reproduced with permission.^[^ ^136^ ^]^ Copyright 2022, John Wiley and Sons. d) Discharge polarization curves and the corresponding power density curves. Reproduced with permission.^[^ ^137^ ^]^ Copyright 2022, John Wiley and Sons. e) Calculated charge density differences for 2L‐Up and FeN_4_. Reproduced with permission.^[^ ^138^ ^]^ Copyright 2020, John Wiley and Sons. f) Half‐wave potential, and g) kinetic current densities (@0.90 V) of the ORR on the as‐prepared PFePc—L/C and Pt/C electrodes. h) X‐band electron paramagnetic resonance (EPR) spectra of PFePc, PFePc—NCS, PFePc—OH, and PFePc—I. i) Calculated Fe 3d orbital energy levels of PFePc and PFePc—L. j) Molecular orbitals of HO—PFePc—L. Reproduced with permission.^[^ ^134^ ^]^ Copyright 2021, John Wiley and Sons.

Recently, Peng et al. reported a special Fe‐based catalyst, in which Fe—N_4_ sites were modulated by axial (subsurface) Fe—O bonds, acquired distinguish ORR catalytic performance (*E*_1/2_ = 0.942 V), and high kinetic current density (*J*_k_ = 39.565 mA cm^−2^) (**Figure** **12**a). The origin of the reinforced ORR activity and role of axial ligands were unveiled by DFT results. The potential limiting step was the final *OH desorption step for most of the models, as shown in Figure 12b, except for the FeN_4_—O model. The optimal free energy changes of the potential limiting step (∆*G* = 0.34 eV) for the FeN_4_—O model were much lower than other structures. Adsorption free energies plots for *OOH, *O, and *OH on the different structural models were shown in Figure 12c, and excellent linear correlations were found in FeN_4_—O, indicating that the introduction of axial O effectively regulated the binding strength of the intermediates.^[^ ^136^ ^]^ Feng and co‐workers utilized axial oxygen ligands to reduce the d‐band center of Zr, endowing Zr sites with a stable local structure and suitable adsorption capacity for intermediates. DFT results illustrated the formation energy of O—Zr—N_4−_ *_n_* C*_n_* was lower than that of Zr—N_4−_ *_n_* C*_n_*, which explained the role O played in ORR process for structural stability of Zr centers. In addition, the high antiaggregation property of Zr enabled ultrahigh loading of 9.1 wt%. The most remarkable thing was that O—Zr—N_4_ was applied in Zn–air battery and achieved a power density of 324 mW cm^−2^, which was the highest reported so far (Figure 12d).^[^ ^137^ ^]^ Well‐designed ON_4_Fe—O—FeN_4_O catalyst with bridge bonded oxygen ligands was constructed by Xing and co‐workers. EXAFS fitting illustrated the presence of both Fe—N and Fe—O with coordination numbers of 4 and 2, respectively. The bridge oxygen modulated the electronic structure of Fe center and greatly reduced the reaction barrier of ORR. At the same time, the formation of double Fe—O bonds endowed the bridge oxygen with higher thermodynamic stability. Charge transfer occurring at the catalyst interface was investigated by PDOS (Figure 12e). The electrons in the d orbital of Fe were injected into O, which reduced the d electrons in the Fe center for weakening the oxygen adsorption and accelerating the ORR process.^[^ ^138^ ^]^ Fe—N_4_—O_2_ catalysts also possessed hexa‐coordinate structure (wherein the Fe single atoms were coordinated to four in‐plane nitrogen atoms and double axial oxygen atoms). Superb ORR performance over a wide pH range was exhibited in both 0.1 m HClO_4_ (*E*_1/2_ = 0.81 V) and 0.1 m KOH (*E*_1/2_ = 0.9 V).^[^ ^139^ ^]^

In addition to the O atom, the Cl atom has also become a popular choice for the axial fifth ligand. Wang and co‐workers fabricated an atomically dispersed FeCl_1_N_4_/CNS (N and S co‐doped carbon) catalyst via thermal‐migrating method. The close interaction between axial Cl atoms and Fe and the distant interaction between S and Fe doped in the substrate together tailored the electronic structure of the active center collectively, giving FeCl_1_N_4_/CNS a moderate charge state and oxygen binding strength.^[^ ^140^ ^]^ In 2021, two excellent research work reported by Wang and co‐workers and Shi and co‐workers focused on axial‐Cl‐coordinated Fe—N_4_ catalyst. While the former found that the introduction of Cl can reduce the valence state of Fe and affect the charge distribution of Fe center,^[^ ^141^ ^]^ the latter compared the changes of catalyst performance before and after dechlorination treatment, and confirmed that the binding of axial Cl atoms significantly enhanced the ORR performance.^[^ ^142^ ^]^ Furthermore, both of their studies illustrated that the fivefold coordination weakened the bonding strength with OH*, thus promoting the optimal adsorption of OH* intermediate.

The above work provided valuable cases for the atom‐level interface modification engineering involved in axial ligands. Nevertheless, a thorough and comprehensive understanding of the mechanism toward axial ligands effecting Fe—N_4_ sites is still needed. Sun and co‐workers selected FePc as the support of the axial ligand, and evaluated the as‐prepared PFePc—ligand (L)/C electrodes with strong field (ethylenediamine > triethylamine > NCS^−^) and weak‐field ligands (OH^−^ > F^−^ > Cl^−^ > Br^−^ > I^−^) in 0.1 m KOH. As shown in Figure 12f,g, the half‐wave potential and current density showed an obvious reinforce with the decrease of the ligand field strength, suggesting clear correlation between the strength of the crystal field and ORR performance. According to the lattice field theory, the ligand has a direct effect on the 3D orbital configuration and the electron spin state of the metal center. In Figure 12h, the major characteristic signals of PFePc—I/C at *g* = 5.91 and 2.09 were attributed to the high‐spin Fe(III) ions. Mössbauer spectroscopy further confirming axial coordination could change the Fe center to a high‐valence and high‐spin state. Furthermore, Fe 3d orbitals in PFePc—L/C were simulated in Figure 12i. The energy level of d*_z_* ^2^ decreased as the field strength of the axial ligands decreased, accompanied by a gradually narrowed energy gap between d*_z_* ^2^ and d*_xz_* d*_yz_*. It is known that the rate determining step (RDS) of PFePc—L/C is the desorption of OH*. The authors simplified the interaction between FePc and OH* to the interaction between Fed*_z_* ^2^ and OH_P_ *_x_*_/P_ *_y_*. DFT analysis (Figure 12j) revealed that the field strength of the axial ligand can regulate the energy level of d*_z_* ^2^. Therefore, the ligands with lower energy of d*_z_* ^2^ bound weakly with OH*, and had higher ORR performance.^[^ ^134^ ^]^

#### 4.3.2 Axial Coordination with N Atoms

**Figure 13:**
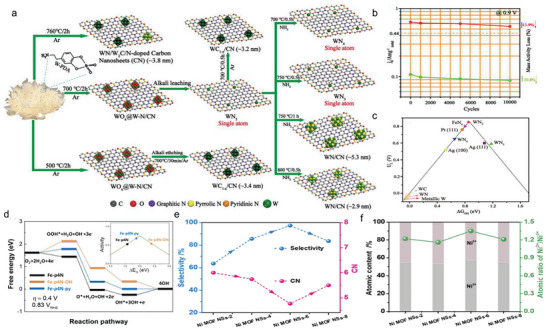
a) Schematic representation of the fabrication of three types of single‐atom SW—N—C complexes with different W—N coordinate numbers. b) The durability of the WN_5_ sample. c) Illustration of the limiting potential (UL) “volcano” as a function of the change in Δ*G*_OH_. Reproduced with permission.^[^ ^144^ ^]^ Copyright 2019, Elsevier. d) Free energy diagrams of ORR processes. Reproduced with permission.^[^ ^145^ ^]^ Copyright 2019, John Wiley and Sons. e) The graph of H_2_O_2_ selectivity and the CN of Ni MOF NSs‐2, 4, 6, and 8. f) The atomic content of Ni^2+^ and Ni^3+^, and the atomic ratio of Ni^2+^/Ni^3+^ in Ni MOF NSs‐2, 4, 6, and 8. Reproduced with permission.^[^ ^146^ ^]^ Copyright 2021, John Wiley and Sons.

Liu et al. defined two kinds of active centers on a six membered carbocyclic ring and found that the overpotential of the five‐coordinated Fe—N_5_/C@G catalyst was lower than FePc or even lower than Pt(111) via DFT calculation. Electrochemical test results showed that the current density of Fe—N_5_/C@G (1.65 mA cm^−2^) was superior to those of FePc (1.04 mA cm^−2^) and Pt/C (1.54 mA cm^−2^) at 0.88 V, which were very well consistent with the DFT calculations.^[^ ^143^ ^]^ Chen et al. prepared W—N_5_ configuration catalysts via tuning the time and atmosphere during pyrolysis process (**Figure** **13**a). W–N_5_ displayed excellent ORR activity at a wide range of pH, and only lost 13.9% of the mass activity over 10 000 cycles (Figure 13b) that surpassed the standard U.S. department of energy making (no more than 40% of the mass activity is lost after more than 10 000 cycles). Just like the volcano plot in Figure 13c, the best d*_z_* ^2^–p orbital hybridization and charge redistribution was contributed to the moderate binding of W—N_5_ to OH*, rather than the overstrong binding of W—N_4_ with OH* or the too weak binding of W—N_3_ with OH*.^[^ ^144^ ^]^ The axial coordination of N atom could accelerate the ORR process, and the energy barrier of OH* to OH^−^ which was the rate‐determining step in Fe—N_5_ catalysts decreased from 0.2 to 0.11 eV (Figure 13d).^[^ ^145^ ^]^

With controllable ratios of metal precursor and organic linkers (*R*_m/l_), Ni‐based catalysts were reported for efficient H_2_O_2_ production. Four catalysts [denoted as Ni MOF nanosheets (NSs)‐2/4/6/8] were investigated whose *R*_m/l_ were 2, 4, 6, and 8. When the *R*_m/l_ grew to 6, the production rate of H_2_O_2_ was 80 mmol g_cat_^−1^ h^−1^ and the selectivity toward H_2_O_2_ reached 98%. XAFS analysis detected that the coordination number of the catalysts increased first and then decreased with the growth of the proportion of precursor (Figure 13e), and those structures were through a process from saturation to partial saturation to destruction. DFT showed that the binding energy of OOH* improved with the decrease of coordination number, meeting variation trend of the H_2_O_2_ selectivity very well. Close to five coordination, Ni MOF NSs‐6 had a higher proportion of Ni^2+^ (Figure 13f) that could achieve more conversions to *β*‐NiOOH, which played an important role in improving the catalytic stability.^[^ ^146^ ^]^

## 5 Summary and Outlook

Since the concept of single‐atom catalysts was proposed, they are always the focus of research because of their well‐defined structures, high atomic utilization efficiency, and controllable coordination environment. Benefitted from these advantages, SACs achieve the efficient atomic economy as well as superb catalytic performance beyond our expectation. However, with the more and more in‐depth identification of active center and coordination environment, planar four‐coordinated M—N_4_ moiety in traditional SACs is no longer considered as the optimal active site.

As determined by recent years’ work, enriching the configuration of metal center or regulating their coordination structure possesses the ability to tailor the electronic structure and further affects the adsorption/desorption strength of the intermediate over atomic active metal centers of SACs during the catalytic process. Distinguishing the type of metal atoms, coordination atom species, and the coordination number, and determining the geometry and electronic structure of SACs could deepen the understanding of the nature in single‐atom active sites.^[^ ^80^, ^81^, ^82^, ^83^, ^84^ ^]^ Hence, in this review, we introduce advanced characterization technology for SACs and systematically illustrate the boosting of oxygen electrocatalysis properties brought by regulating active center and the first coordination sphere of SACs. Owing to these beneficial regulation, many inherent properties in SACs, such as the metal loading, electronic structure, reaction path, and catalytic activity were significantly improved.

Up to now, the exploration of SACs has become increasingly mature. In the future, SACs need to “walk” out of the laboratory to cater for industry demand and the development of national energy strategy. Herein, we propose five prospects.

1) There is a seesaw effect between the metal sites and their support. During the pyrolysis, too strong interaction between them will trigger Ostwald maturation. However, weak binding to the support makes SACs very unstable, where the migration and agglomeration phenomenon will become inevitable. In actual industrial production, catalysts generally are required to work for months under high temperature and high pressure environment in order to pursue the maximum economic profits. Therefore, it is quite necessary to develop the catalysts with long‐term thermodynamic stability and activity.
2) It is well‐known that the production and commercialization of energy devices are significantly dependent on their cost competitiveness. Increasing catalyst loading is the crux to improve the output power and lower the cost of electrochemical devices. Nevertheless, in order to avoid the excessive surface energy of single atoms and the formation of metal nanocrystals, most SACs have relatively low loading, leading to unsatisfactory overall catalytic activity. In the future, more efforts are needed in the development of advanced material preparation method to achieve high loading (preferably above 3 wt%) of metal sites in targeted atomic catalysts.
3) The change of coordination structure in SACs is noteworthy. A valuable case is that the peptides and proteins at the center of the enzyme have a chiral structure and are kept in motion. They can achieve a higher effective collision probability during catalysis, which greatly improves the selectivity of the reaction and reduces the activation energy of the reaction. The rich adjustable characteristics of SACs may bring hope to solve the high energy consumption problem in the traditional catalytic process.
4) The exploration of the activity and mechanism of SACs requires the development of in situ characterization techniques. In situ/operando XAFS and FTIR have the ability to monitor the dynamic process of the evolution of active sites at atomic or molecular level. Plenty of clues for the structural evolution, which are highly related to the coordination geometry, oxidation state, and related reaction intermediates, could be gained in real time during electrocatalytic process. Undoubtedly, it will deepen our comprehensive understanding of the essential electrocatalytic mechanism for SACs toward electrocatalytic performance improvement. Thus, in situ/operando characterization techniques should be widely promoted.
5) When the coordination structure of a catalyst is confirmed, researchers are eager to know which reaction the catalyst is best for and what its potential effect on catalytic behavior might be. Therefore, in the future, we need to combine artificial intelligence and machine learning to develop technologies with data statistics and rapid analysis functions, which will speed up the efficiency of experimental analysis and design.

Looking ahead, the development of SACs still faces many challenges. However, atom‐level interface modification engineering has provided some novel insights into SACs’ revolution, and would build the bridge for the atomic catalysts toward more extensive application.

## Conflict of Interest

The authors declare no conflict of interest.
